# LeAf Trauma- an intersectoral prospective multicenter study assessing quality of life and return to work after majortrauma–study protocol

**DOI:** 10.1371/journal.pone.0312320

**Published:** 2024-11-13

**Authors:** Katharina Fetz, Gina Grimaldi, Dan Bieler, Anne Neubert, Carina Jaekel, Christine Hoefer, Elisabeth Schwojer, Stefanie Bartha, Jean-Jacques Glaesener, Lars Becker, Lisa Wienhoefer, Rolf Lefering, LeAf-Trauma Study Group

**Affiliations:** 1 Institute for Research in Operative Medicine, Witten/Herdecke University, Cologne, Germany; 2 Department of Trauma Surgery, Otto-Von-Guericke University Magdeburg, Magdeburg, Germany; 3 Department for Trauma Surgery and Orthopaedics, Reconstructive and Hand Surgery, Burn Medicine, Germany Armed Forces Central Hospital Koblenz, Koblenz, Germany; 4 Department of Orthopedics and Traumatology, University Hospital and Medical Faculty Duesseldorf, Heinrich-Heine-University Duesseldorf, Duesseldorf, Germany; 5 AUC-Academy for Trauma Surgery, Munich, Germany; 6 Department of Trauma, Hand, and Reconstructive Surgery, University Hospital Essen, University of Duisburg-Essen, Essen, Germany; Public Library of Science, UNITED KINGDOM OF GREAT BRITAIN AND NORTHERN IRELAND

## Abstract

With increasing survival rates, the functional outcome and quality of life of trauma patients are gaining more importance. Survivors suffer from chronic pain, psychosomatic disorders, and unemployment as well as increased post-traumatic morbidity, which can lead to an impaired quality of life. So far, the TraumaRegister DGU^®^ records patient data during in-hospital treatment. In this study severely injured patients after major trauma are assessed when discharged from hospital, as well as 6, 12 and 18 months after trauma. The aim is to document cross-sector patient pathways and to identify and quantify the factors influencing the health-related quality of life (hrQoL) and the return to work (RTW), using patient-reported experience measures (PREM) and patient reported outcome measures (PROM). Patients are recruited in certified trauma centers of the German Society for Trauma Surgery (DGU). This study protocol describes the methodology of the prospective multicentre study of LeAf Trauma. Translation of the results will be implemented by using the network structures of the German Society for Trauma Surgery (DGU) for the treatment of patients with major trauma.

## Introduction

Major trauma refers to multiple, severe, and often life-threatening injuries sustained by an individual because of an accident or violence. These injuries may involve one or more body systems and organs and often require immediate medical attention. Major trauma can result from various incidents, including motor vehicle accidents, falls from significant heights, industrial accidents, assaults or sports injuries. In Germany, approximately 30,000 people suffer from a severe trauma each year (annually reports TraumaRegister DGU^®^, national German Trauma Registry of the German Society for Trauma Surgery) [1].

Due to advances in the preclinical and clinical treatment, the mortality of severely injured patients has been significantly reduced. Data of the TraumaRegister DGU^®^ from 2023 report a mortality rate of 13,1% [1]. With increasing survival rates, the functional outcome and quality of life of trauma patients are gaining more importance. Survivors suffer from chronic pain, psychosomatic disorders, and unemployment [2, 3], as well as increased post-traumatic morbidity [4–6] which can lead to an impaired quality of life.

In Germany, 60% of severely injured patients are aged 18–65 years and therefore in the center of their working age. Returning patients to work is important to promote their quality of life and social participation, and to reduce the socio-economic burden on society caused by incapacity to work. With a statutory retirement age of currently 66 years, inability to work can result in a financial provision gap of several decades. According to surveys by Häusler et al. over 20 years ago, an accident with multiple injuries already cost an average of EUR 500,000 [7] with around two thirds of the costs arising from short- and long-term loss of work. The following predictors of clinical treatment for an inability to work after severe trauma have already been identified: patient age, injury severity, traumatic brain injury, duration of intensive care treatment, psychological burden, subjective perception of the injury, and patient education level [8] It is yet not fully understood, which mechanisms cause patients to remain unable to work or hold them back to return to work if their work ability is regained. Possible causes could also be of organizational nature, for example delays in rehabilitation or the financing of retraining.

The acute medical treatment in a trauma center is frequently followed by rehabilitation therapy. The success of this therapy is not determined by the duration alone, but by the quality of the rehabilitation [9]. Organizational restrictions and barriers of the health care system often led to a delay in the onset of rehabilitation therapy, also called as gap in the rehabilitation process. It is currently unclear what rehabilitation interventions and aids are needed on average after a severe trauma.

There is a need to investigate the reality of severely injured individuals throughout the entire recovery process and across sector boundaries, integrating the patient experiences and needs along the cross-sectoral pathway. Therefore, the aim of the LeAf Trauma (health related quality of life and return to work after severe trauma)–project is to fill an existing knowledge gap by improving the understanding of cross-sector patient pathways and identifying and quantifying the factors influencing the quality of life and a timely return to work of patients after severe trauma.

## Methods

The LeAf Trauma project consists of two study arms, a prospective multicenter study and a retrospective routine data analysis of AOK (Allgemeine Ortskrankenkasse, German General Health Insurance) ‐ health insurance data. This study protocol describes the methodology of the prospective multicentre study. The secondary routine data analysis of the AOK health insurance data is described elsewhere (AOK et al).

An overview of study periods s given in the SPIRIT schedule in supporting information (S1 File) along with the SPIRIT checklist. A written lead ethical vote was obtained from the ethical committee of the Heinrich-Heine-University Düsseldorf, Germany (study number 2022–2029). The structure of the prospective study arm includes over 40 study clinics for which ethical vote had to be extended. Hence, with this lead ethical vote we applied an ethical approval at several universities and regional chambers of physicians in Germany. Several other ethical committees followed the lead ethical vote and granted us their ethical approval. For other clinics, we had to apply for an ethical vote at their university or at the regional chambers of physicians again. A full list universities and regional chambers of physicians that granted us an ethical approval for the prospective study arm is included in S2 File. The study was registered at the WHO clinical trial registry (registration number: DRKS00028841).

In the prospective study, the entire course of treatment and outcomes of a group of severely injured patients will be documented across sector boundaries for a period of 18 months. Additionally, the individual patient experiences will be assessed using patient-reported experience measures (PREM) and patient-reported outcome measures (PROM). PREMs are tools used to gather feedback from patients about their experiences during the recovery process, they are specifically concerned with the patient’s perspective on the process of care [10]. PROMs on the other hand assess the impact of healthcare services on a patient’s health status or quality of life. However, primary outcomes in this study are health-related quality of life and return to work. Whereas the other examined items work as predictors influencing the primary outcomes. Many examined predictors are patient-reported experience measures, an overview of used PREMS can be found in Table 1.

**Table 1:** PREM and PROM used in prospective LeAf Trauma study.

PREM ‐ Patient Reported Experience Measures	PROM ‐ Patient Reported Outcome Measures
social support	
social responsibility	
self-appraisal of injury severity	
circumstances of accident	
perception of acute care treatment	
rehabilitation	
post hospital treatment	
	health-related quality of life
	return to work
	general treatment evaluation

PREM: patient-reported experience measures, PROM: patient-reported outcome measures

The chosen study design aims to examine the relationships between the general background of patient (e.g. age, pre-existing disease), injury (type, severity), medical intervention (initial care, rehabilitation, physiotherapy, etc.) and system (access to and availability of medical intervention) and to identify the multifactorial predictors for unfavorable and favorable outcomes. The main hypotheses are:

1. There are influenceable predictors for returning to work in the cross-sectoral course of treatment.
2. These factors also contribute to the patient’s post-traumatic condition (health-related quality of life)
3. PREMs help to identify such factors and to develop recommendations and measures to improve patient care.

### Measurement timepoints

Patients are assessed when discharged from hospital, 6, 12 and 18 months after trauma. Prior to any questioning, written consent has been obtained by every patient. The baseline questionnaire is available as a paper-pencil version and will be handed to the patients by the clinic staff shortly before the end of the inpatient stay, together with the information documents and various recruitment materials (flyers, brochures). In addition, medical data will be collected on a separate form at baseline by the study physician. All information is then checked for completeness by the clinical trial staff and entered the trial registry on the AUC registry platform.

In contrast to the baseline survey, at the time of the follow-up survey 6, 12 and 18 months after the accident, the patients are generally no longer inpatients at the study clinic but are in the individual patient pathway. For this reason, the follow-up is carried out using web-based questionnaires, as other studies have already demonstrated the success of recruiting and following up large cohorts using this approach [11–13].

**Fig 1:**
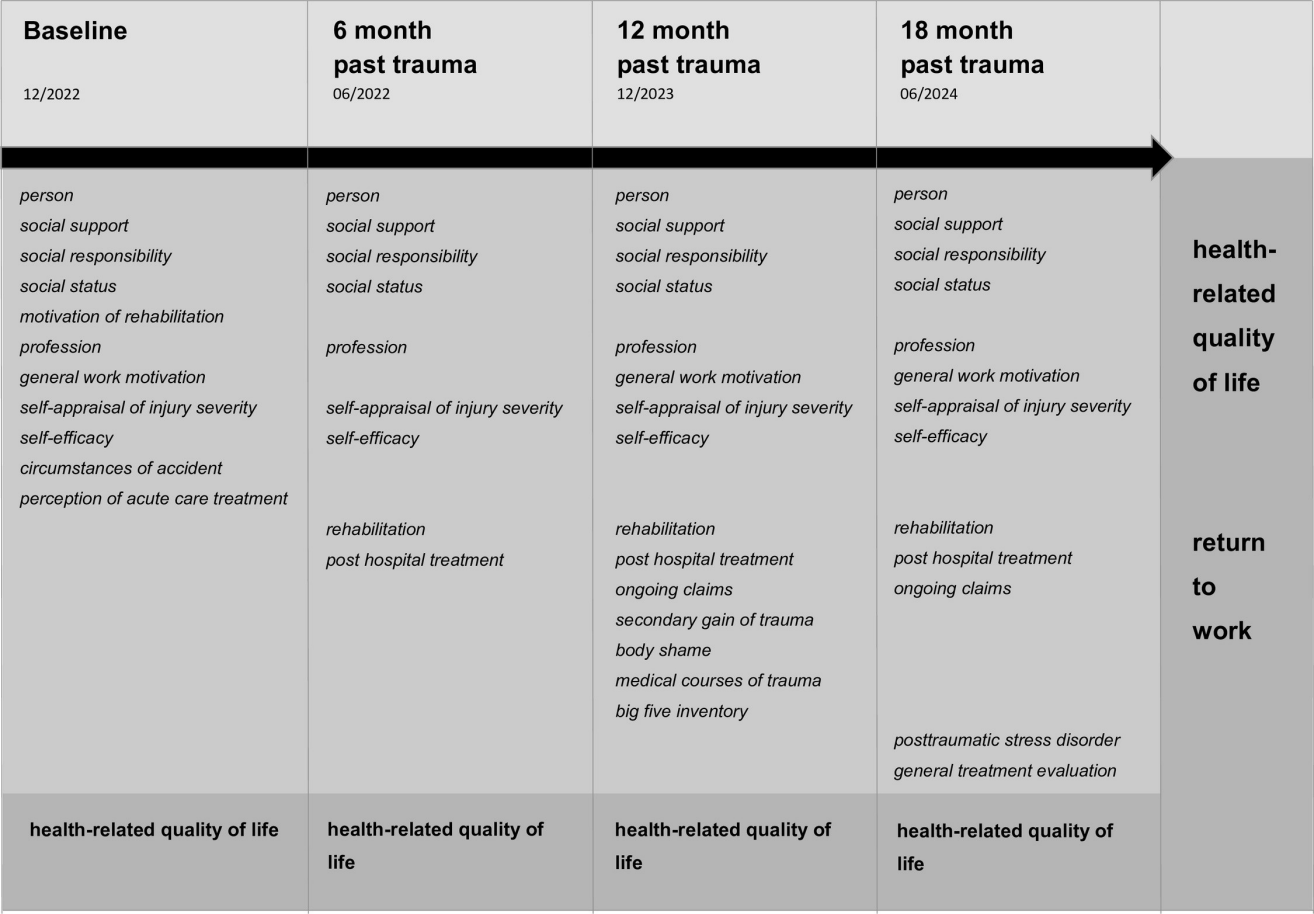
Overview of measurement time, predictors and outcome parameters of LeAf Trauma. Light grey: measurement time points, grey: predictor, dark grey: outcome.

The study clinics are informed of the AUC two weeks before each patient’s follow-up date and receive an email template to forward to the patients. This email contains the access link and individual access code to the online survey, which will be conducted using LimeSurvey, an open-source online survey application [14]. The survey takes between 30 and 40 minutes to complete. Depending on the type of response, follow-up questions will be shown or hidden, thus reducing the content to what is individually relevant. If the patient does not respond within two weeks, a telephone or written reminder will be sent. During the phone call, the study staff offers assistance in completing the questionnaire. If patients refuse to complete the online survey via LimeSurvey, the study clinics will provide a paper-based follow-up questionnaire by post. The data from the paper questionnaire will then be entered into LimeSurvey by the study staff. Inclusion of patients started in 12/2022, the last baseline assessment will be included in 09/2024. The measurement times and targets identified are shown in Fig 1.

### Selection and development of measures

Primary endpoints of the prospective study are health-related quality of life (hrQoL) and return to work (RTW). Potential predictors for these endpoints have been identified based on a literature research, experts discussions of the interdisciplinary research team, and qualitative interviews with trauma patients and experts in the field of trauma (Neubert et al., in prep). Whenever possible, validated instruments are used. If no validated measure for a specific aspect was available, new items have been developed by means of an interdisciplinary consensus process informed by experts in the field of research methodology and test construction. All items for each assessment period have been pre-tested with trauma patients to ensure comprehensibility, feasibility and to estimate required time to complete the questionnaire. In addition, questionnaires of 6-month and 12-month assessments have been discussed with a group of experts in Germany to rate the pertinence and importance of the used items in semi-structured interviews. The item sets for each measurement timepoint are illustrated in Table 2.

**Table 2:** Itemset of the prospective LeAf Trauma study.

				baseline	follow up 6	follow up 12	follow up 18
subject	instrument	dimension	item				
medical details	ICD-10-Code [20]		1 item	x			
	*self-constructed questions*		pre-existing illness	x			
			health insurance	x			
			pre-trauma pain therapy	x			
			hospital discharge location	x			
	Barthel-Index, Assessment of basic activities of daily living [21]		10 items	x			
	Trauma-Reha-Score Screening [22]		9 items	x			
	Trauma-Register data [23]		112 items	x			
person	body mass index		2 items	x	x	x	x
	*self-constructed questions*		age	x			
			sex	x			
			marital status	x	x	x	
			immigration history	x			
			unemployment pre trauma	x			
social support	Oslo Social Support Scale [24]		3 items	x	x	x	x
	*self-constructed question*		relationship	x	x	x	x
			surrounding: 2 items		x		
social responsibility	*self-constructed questions*		number of persons per household	x	x	x	x
			principal earner of household	x	x	x	x
			trauma related financial problems				x
social status	Winkler-Index [25, 26]		household income	x	x	x	x
			educational attainment	x			
motivation of rehabilitation	PARMEO, Patient questionnaire for assessing rehabilitation motivation [19]	level of information	3 items	x			
		scepticism	3 items	x			
profession	ISCO, international scale of occupation [27]		1 item	x			
	REFA (German Committee for Determining Working Hours), Classification of heavy workload [28]		1 item	x	x	x	x
	*self-constructed questions*		employment status	x			
			occupation	x			
general work motivation	DIAMO, Diagnostic instrument for work motivation [29]	attitude regarding work	6 items	x		x	x
		goal-inhibition	6 items	x			
		goal-activity	6 items	x			
self-appraisal of injury severity	*self-constructed questions*		Likert scale: subjective evaluation of trauma	x			
			Subjective evaluation of returning to work	x	x	x	
			subjective possibility to avoid accident	x			
self-efficacy and resilience	ASKU, German General Self-Efficacy Short Scale [30]		3 items	x	x	x	x
	Re-Re Scale Resistance-Regeneration Orientation Scale [31]		10 items		x		
health related quality of life	European Quality of Life 5 Dimensions 3 Level Version [32]		5 items	x	x	x	x
	TOP, Trauma Outcome Profile [33]	anxiousness	4 items	x	x	x	x
		depression	4 items	x	x	x	x
		posttraumatic stress disorder	4 items	x	x	x	x
		pain	15 items	x	x	x	x
		body functions	15 items	x	x	x	x
		social interaction/ financial problems	4 items		x	x	x
		activity of daily living (ADL)	4 items		x	x	x
		mental function	4 items		x	x	x
		body image	1 item		x	x	x
		overall satisfaction	1 item		x	x	x
	Short-Form-Health-Survey-12 [34]		12 items		x	x	x
	*self-constructed questions*		Likert scale: self-appraisal of health and wellness	x	x	x	x
			sleep quality		x	x	x
			spirituality		x	x	
			sexual life				x
circumstances of accident	*self-constructed questions*		accident framework	x			
			accident recall	x			
			presence of accident opponent	x			
			ongoing claims		x	x	x
			subjective or objective fault	x			
perception of acute care treatment	*self-constructed questions*		evaluation of preclinical treatment	x			
			evaluation of emergency department treatment	x			
			Information level regarding treatment	x			
			Likert scale: pain treatment	x			
return to work	*self-constructed questions*		health insurance		x	x	
			ongoing work incapacity		x	x	x
			occupational rehabilitation		x	x	x
			change of income or work hours		x	x	x
			occupational redeployment		x	x	x
			current occupation		x	x	x
			commute		x	x	x
			support by employer			x	x
rehabilitation	*self-constructed questions*		current residence		x	x	x
			information level of treatment		x	x	x
			rehabilitation outpatient or inpatient		x	x	x
			direct transfer to rehabilitation		x	x	x
			length of rehabilitation		x	x	x
			evaluation of exercises, tailored rehabilitation program		x	x	x
			participation opportunity of exercises		x	x	x
			subjective recovery after rehabilitation		x	x	x
			evaluation of discharge-management		x	x	
			evaluation of rehabilitation manager		x	x	x
secondary gain of trauma	FPTM ‐ Questionnaire for assessing psychotherapy motivation [35]	symptom-focused attention from others	6 items			x	
body shame	*self-constructed questions*		7 items			x	
posthospital treatment	*self-constructed questions*		evaluation of discharge management		x	x	x
			helplessness after discharge		x	x	x
			information level at time of discharge		x	x	
			ongoing physiotherapy after hospital treatment		x	x	x
			Speciality/profession of treating physician		x	x	x
			evaluation of treatment coordination by physician		x	x	x
			Likert-scale: evaluation of pain therapy		x	x	x
			ongoing psychological, psychiatric or psychotherapeutic treatment		x	x	x
			access to post hospital paramedical treatment		x	x	x
			quality of contact with authorities regarding trauma consequences		x	x	x
			evaluation of hospital treatment			x	
			evaluation of post-hospital treatment			x	
medical courses of trauma	*self-constructed questions*		complications during the healing processFormularbeginnFormularende		x	x	x
			further operations after discharge from hospital		x	x	x
			ongoing treatment regarding accident				x
personality	Big Five Factor Model [36]		10 items			x	
PTSD ‐ posttraumatic stress disorder	International trauma questionnaire [37]		9 items				x
treatment evaluation	*self-constructed questions*		overall evaluation of treatment				x
			evaluation of general life situation after trauma				x
			change on outlook on life after trauma				x
			problems occurring during post hospital care				x

### Patient recruitment and data collection

Patients are recruited in certified trauma centers of the German Society for Trauma Surgery (DGU). The trauma centers need to be a certified DGU Trauma Centers, that treat at least 80 severely injured patients per year according to the inclusion criteria of the TraumaRegister DGU^®^.

The participating trauma centers received comprehensive training on study procedures and follow-up assessments, recruitment, administration, tracking, and follow-up assessments are carried out by the respective trauma center. These trauma centers receive a compensation of maximal 660€ per patient staggered according to the number of successfully documented follow-ups. All data are collected exclusively in pseudonymized form in a web-based study database. The list of pseudonyms is managed by the participating trauma centers. After informed consent, baseline data are collected before the patient is discharged. Follow-up assessments are conducted at 6, 12,and 18 months after trauma, links for LimeSurvey access are sent by the trauma center. On special request if online access is not available for the patient, paper-pencil documents are sent.

### Inclusion and exclusion criteria

**Fig 2:**
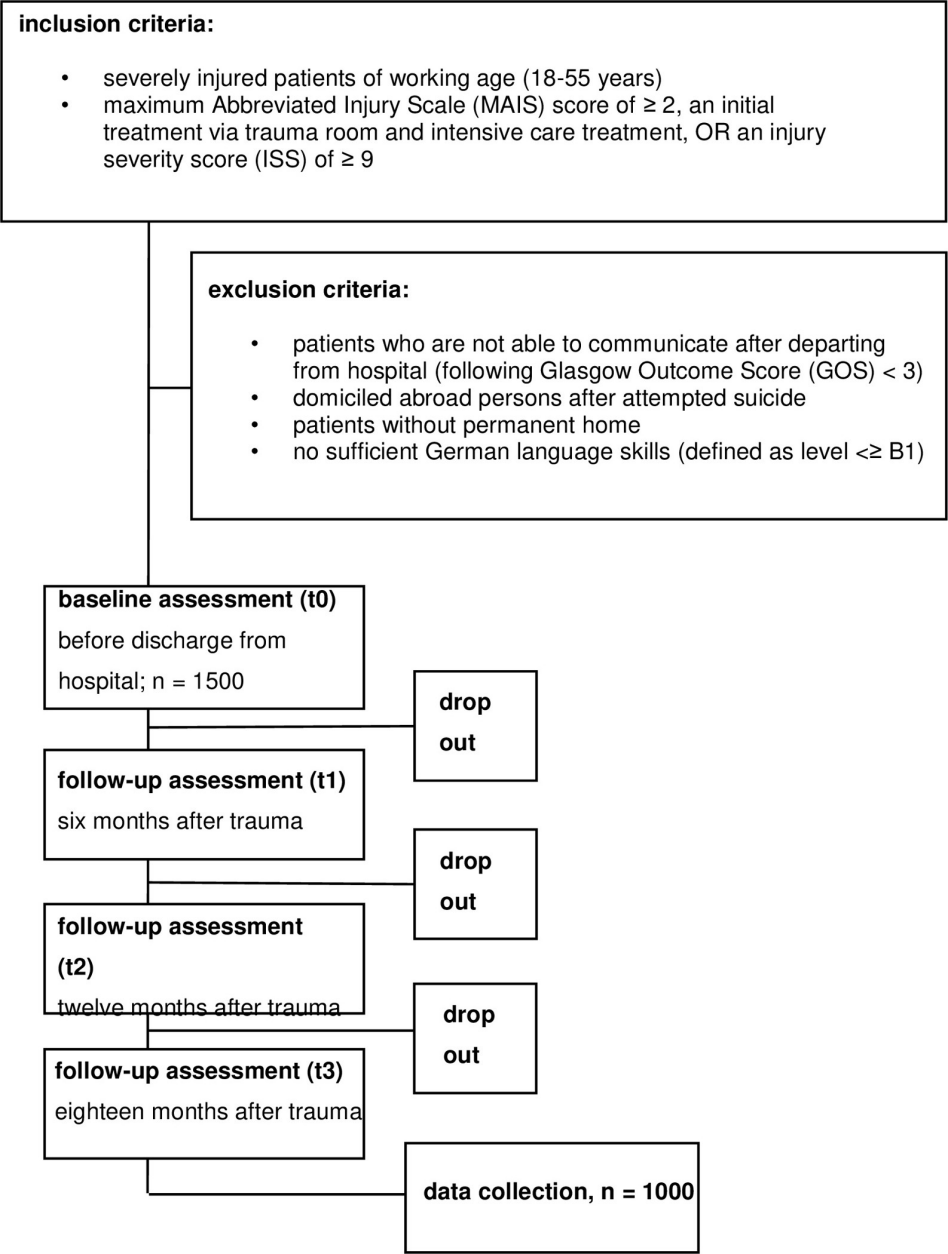
Inclusion- and exclusion criteria with expected drop-out rates.

For the prospective study, patients are recruited from12/2022 to09/2024. The initial inclusion criteria are severely injured patients of working age (18–55 years) with a Maximum Abbreviated Injury Scale (MAIS) score of ≥ 2, an initial treatment via trauma room and intensive care treatment, OR an injury severity score (ISS) of ≥ 9. Exclusion criteria are patients who are not able to communicate after discharge from hospital, following Glasgow Outcome Score (GOS) <3 [15], domiciled abroadpersons after attempted suicide, patients without permanent home or no sufficient German language skills (defined as level <≥ B1,European Reference).In 2023 the inclusion criteria have been amended to foster inclusion rates. The inclusion criteria and process are shown in Fig 2.

### Sample size analysis

Since severe injuries can occur in different regions of the body, the present cohort is expected to be heterogeneous. Therefore, the collective must be large enough to examine socioeconomic and psychosocial factors in addition to patient- and injury-specific subgroup analyses (e.g. injury of lower extremity).With a total sample size of n = 1000 evaluable cases (with complete follow-up), a prevalence of 10% of an outcome criterion could be given with an accuracy of +/- 2% (95% confidence interval), a prevalence of 20% with +/- 2.5%, and a prevalence of 50% with +/- 3%. In subgroups of 300 cases, there will still be an accuracy of 3.5% / 4.5% / 5.5% for the prevalence estimations.

For the multivariate analyses of predictors, a number of 5–10 patients with a respective outcome (RTW, poor hrQoL) should be available for each predictor. If approx. 30% of the patients did not return to work, approx. 30 risk factors could be examined for the entire collective. A response rate of about 50–66% is assumed [16], of which it is expected that25-30% will not complete all follow-up assessments. According to empirical values from the TraumaRegister DGU^®^, an average of 120 severely injured patients (MAIS ≥ 3) are treated per year in a typical level 1 trauma center in Germany. After excluding the deceased (approx. 10%), around 100 patients remain (still excluding traumatic brain injuries). With a participation rate of 50% (n = 50) and a loss to follow-up of one third, n = 35 patients per year could be recruited per trauma center. Thus 30 participating level 1 trauma centers are necessary to obtain 1000 evaluable patients in a one-year inclusion phase. A screening log of potential study participants is kept by each participating trauma center to estimate a selection bias. In addition, the patient characteristics will be compared with the collective of the TraumaRegister DGU^®^, using the same inclusion criteria (representativeness).

The gold standard for predictor testing in prognosis studies is a defined cohort with a high follow-up rate. It is aimed to achieve this goal by recruitment of many study trauma centers.

### Statistical analysis

Data will be exported from the AUC registry platform [17] and LimeSurvey [14] transferred to IBM SPSS statistics for analysis. Data will be checked for plausibility and missing values prior to analysis. To ensure data completeness, participating hospitals are informed to complete the data in case of missing clinical data. If the patient reported follow-up questionnaire is incomplete, the corresponding hospital gets a query and will contact the patient up to two more times in order to encourage the patient to complete the form. All Statistical analyses are performed using IBM SPSS and R [18].

Descriptive statistics include patients’ clinical progression, description of recovery regarding RTW and hrQoL over three follow-up timepoints. Predictors for RTW are analyzed by using logistic regression analysis for the dichotomous criterion return to work (yes/no). Predictors will be categorized into the categories: patient, occupation, pre-hospital, hospital, post-acute therapy, presence of psychological problems. The period until RTW will be plotted in Kaplan-Meyer curves for descriptive analysis. A multivariate cox regression will be used to analyze predictors for latency of return to work. HrQoL, PREMs and PROMs are reported descriptively for all three follow-up time points. In case of partial missing follow-ups, hrQoL will be estimated based on those with complete follow-up (responders). Responders and non-responders (no follow-up) will be compared. Additionally, subgroup analyses are performed at each follow-up timepoint. The relation of hrQoL and RTW is assessed by means of correlation analysis.

## Discussion

This study represents the first comprehensive prospective examination of a large cohort of severely injured patients up to 18 months post-trauma in certified DGU Trauma Centers in Germany and Austria. Qualitative interviews conducted in preparation of the prospective study enable the targeted identification of previously unknown predictors of hrQoL and RTW. By collecting PREM and PROM the patient pathways including post-hospital care and rehabilitation of polytraumatized patients can be thoroughly illustrated, and their impact on hrQoL and RTW is assessed. The results gained of each measure point are considered for the subsequent follow up measure. The follow up questionnaires are thus dynamically designed and consist of longitudinal and single point assessments. This allows the inclusion of further identified predictors in the final 18 months follow-up assessment. Through the retrospective study arm initial consolidations of ICD-10coding into useful trauma constellations is made. Subsequently, the healthcare pathway of polytraumatized patients and the time until return to work is achieved can be retrospectively depicted and compared in subgroups. The goal of the study is to identify predictors for the hrQoL and RTW of polytraumatized patients and, in the long term, improve the care of severely injured patients. Translation of the results will be implemented by using the network structures of the German Society for Trauma Surgery (DGU) for the treatment of patients with major trauma. Measures for patient empowerment will be undertaken in the hospitals of acute care to foster the chances for a preferable pathway along the cross sectoral treatment.

### Limitations

The prospective study focuses on patient-reported experience- and outcome-measures. An objectifiable clinical examination of patients is not performed in the follow-up assessments. Thus, healthcare services such as rehabilitation or outpatient follow-up treatment can only be analyzed from the patient’s perspective. This information is therefore obtained from the retrospective part of the study. However, the subgroups of the prospective and retrospective arms cannot be easily compared with each other.

## Supporting information

S1 File SPIRIT schedule and checklist. (PDF)

S2 File List of study clinics and ethical approval. (PDF)

S3 File Patient information and consent form_translation. (PDF)

S4 File Study protocol ‐ original German. (PDF)

S5 File Study protocol English translation. (PDF)

S6 File WHO trial registration. (PDF)

S7 File LeAf Trauma item set English translation. (PDF)

## Abbreviations

AOK: Allgemeine Ortskrankenkasse, German General Health Insurance
ASKU: Allgemeine Selbstwirksamkeitsskala, German General Self-Efficacy Short Scale
Barthel-Index: Assessment of basic activities of daily living
BFI-10: Big Five Inventory ‐ 10
BMI: Body mass index
DIAMO: Diagnostic instrument for work motivation
DGU: Deutsche Gesellschaft für Unfallchirurgie (German Society for Trauma Surgery)
EQ-5D: European Quality of Life 5 Dimensions 3 Level Version
FPTM: Questionnaire for assessing psychotherapy motivation
GOS: Glasgow Outcome Scale
hrQoL: health-related Quality of Life
ISCO: International Scale of Occupation
ITQ: International Trauma Questionnaire
PARMEO: Patientenfragebogen zur Erfassung der Rehamotivation(english: Patient questionnaire for assessing rehabilitation motivation)
PREM: Patient Reported Experience Measure
PROM: Patient Reported Outcome Measure
PTSD: Post-traumatic stress disorder
REFA: Reichsausschuß für Arbeitsermittlung, German Committee for Determining Working Hours
Re-Re Scale: Resistance-Regeneration Orientation Scale
RTW: Return to Work
SF12: Short-Form-Health-Survey-12
TR-DGU: TraumaRegister DGU^®^of the German Society for Trauma Surgery
TOP: Trauma Outcome Profile
