# Correction: LeAf Trauma- an intersectoral prospective multicenter study assessing quality of life and return to work after majortrauma–study protocol

**DOI:** 10.1371/journal.pone.0332632

**Published:** 2025-09-16

**Authors:** The PLOS One Staff

The words “major and trauma” are misspelled in the article title. The correct title is: LeAf Trauma- an intersectoral prospective multicenter study assessing quality of life and return to work after major trauma–study protocol. The correct citation is: 1.Fetz K, Grimaldi G, Bieler D, Neubert A, Jaekel C, Hoefer C, et al. (2024) LeAf Trauma- an intersectoral prospective multicenter study assessing quality of life and return to work after major trauma–study protocol. PLoS ONE 19(11): e0312320. https://doi.org/10.1371/journal.pone.0312320.

The ORCID iD is missing for the first author. Please see the author’s ORCID iD here:

- Author Katharina Fetz’s ORCID iD is: 0000-0002-2391-2243

(https://orcid.org/0000-0002-2391-2243).

The 13^th^ author’s name is spelled incorrectly. The correct name is: LeAf Trauma Study Group.

There are errors in the author affiliations. The correct affiliations are as follows:

Katharina Fetz^1, 2, 3^, Gina Grimaldi^4^, Dan Bieler^5,6^, Anne Neubert^6^, Carina Jaekel^6^, Christine Hoefer^7^, Elisabeth Schwojer^7^, Stefanie Bartha^7^, Jean-Jacques Glaesener^7^, Lars Becker^8^, Lisa Wienhoefer^8^, Rolf Lefering^1^, LeAf Trauma Study Group

**1** Institute for Research in Operative Medicine, Witten/Herdecke University, Cologne, Germany, **2** Department of Anaesthesiology and Operative Intensive Care, Cologne-Merheim Medical Center, university hospital of Witten/Herdecke University, Ostmerheimer Str. 200, Cologne 51109, Germany, **3**

Chair of Research Methodology and Statistics, Witten/Herdecke University, Witten, Germany **4** Department of Trauma Surgery, Otto-Von-Guericke University Magdeburg, Magdeburg, Germany, **5** Department for Trauma Surgery and Orthopaedics, Reconstructive and Hand Surgery, Burn Medicine, Germany Armed Forces Central Hospital Koblenz, Koblenz, Germany, **6** Department of Orthopedics and Traumatology, University Hospital and Medical Faculty Duesseldorf, Heinrich-Heine-University Duesseldorf, Duesseldorf, Germany, **7** AUC-Academy for Trauma Surgery, Munich, Germany, **8** Department of Trauma, Hand, and Reconstructive Surgery, University Hospital Essen, University of Duisburg-Essen, Essen, Germany.

The word LeAF Trauma appears incorrectly throughout the article. The correct word is LeAf Trauma.

In the Methods section, there is an error in the first, third and seventh sentence of the second paragraph.

The correct first sentence is: An overview of study periods is given in the SPIRIT schedule in supporting information (S1 File) along with the SPIRIT checklist.

The correct third sentence is: The structure of the prospective study arm includes over 40 study clinics, for which ethical vote had to be extended.

The correct seventh sentence is: A full list of universities and regional chambers of physicians that granted us an ethical approval for the prospective study arm, is included in S2 File.

In the Measurement timepoints subsection of the Methods, there is an error in the fifth sentence of the first paragraph. The correct sentence is: All information is then checked for completeness by the clinical trial staff and entered to the trial registry on the AUC registry platform.

In the Measurement timepoints subsection of the Methods, there is an error in the first sentence of the second paragraph. The correct sentence is: In contrast to the baseline survey, at the time of the follow-up survey 6, 12 and 18 months after the accident, the patients are generally no longer inpatients at the study clinic, but are in the individual patient pathway.

In the Measurement timepoints subsection of the Methods, there is an error in the ninth sentence of the third paragraph. The correct sentence is: Inclusion of patients started in 12/2022, the last baseline assessment will be included in 11/2024.

In the Selection and development of measures subsection of the Methodology, a reference is incorrect in the second sentence of the paragraph.

The correct sentence is: Potential predictors for these endpoints have been identified based on a literature research, experts discussions of the interdisciplinary research team, and qualitative interviews with trauma patients and experts in the field of trauma (Neubert et al., 2024).

The reference is: Neubert A, Hempe S, Jaekel C, Gaeth C, Spering C, Fetz K, Windolf J, Kollig E, Bieler D; LeAf-Trauma-Group. Lived experiences of working-age polytrauma patients in Germany - A qualitative Analysis. Injury. 2024 Oct 16:111938. doi: 10.1016/j.injury.2024.111938. Epub ahead of print. PMID: 39477709.

In the Patient recruitment and data collection subsection of Methods, there is an error in the second sentence of the first paragraph. The correct sentence is: The trauma centers need to be certified DGU Trauma Centers, that treat at least 80 severely injured patients per year according to the inclusion criteria of the TraumaRegister DGU.

Figs 1, 2 and Table 2 were formatted incorrectly. Please see the correct Figs 1, 2 and Table 2 here.

**Table 2:** Itemset of the prospective LeAf Trauma study.

				base line	follow up 6	follow up 12	follow up 18
subject	instrument	dimension	item				
medical details	ICD-10-Code (18)		1 item	x			
	*self-constructed questions*		pre-existing illness	x			
			health insurance	x			
			pre-trauma pain therapy	x			
			hospital discharge location	x			
	Barthel-Index, Assessment of basic activities of daily living (19)		10 items	x			
	Trauma-Reha-Score Screening 7/1/2024 1:00:00 PM		9 items	x			
	Trauma-Register data (20)		112 items	x			
person	body mass index		2 items	x	x	x	x
	*self-constructed questions*		age	x			
			sex	x			
			marital status	x	x	x	
			immigration history	x			
			pre-existing illness	x			
			unemployment pre trauma	x			
social support	Oslo Social Support Scale (21)		3 items	x	x	x	x
	*self-constructed question*		relationship	x	x	x	x
			surrounding: 2 items		x		
social responsibility	*self-constructed questions*		number of persons per household	x	x	x	x
			principal earner of household	x	x	x	x
			trauma related financial problems				x
social status	Winkler-Index (22);(23)		household income	x	x	x	x
			educational attainment	x			
motivation of rehabilitation	PARMEO, Patient questionnaire for assessing rehabilitation motivation (17)	level of information	3 items	x			
		scepticism	3 items	x			
profession	ISCO, international scale of occupation (24)		1 item	x			
	REFA (German Committee for Determining Working Hours), Classification of heavy workload (25)		1 item	x	x	x	x
	*self-constructed questions*		employment status	x			
			occupation	x			
general work motivation	DIAMO, Diagnostic instrument for work motivation (26)	attitude regarding work	6 items	x		x	x
		goal-inhibition	6 items	x			
		goal-activity	6 items	x			
sellf-appraisalof injury severity	*self-constructed questions*		Likert scale: subjective evaluation of trauma	x			
			Subjective evaluation of returning to work	x	x	x	
			subjective possibility to avoid accident	x			
self-efficacy and resilience	ASKU, German General Self-Efficacy Short Scale (27)		3 items	x	x	x	x
	Re-Re Scale Resistance-Regeneration Orientation Scale (28)		10 items		x		
health related quality of life	European Quality of Life 5 Dimensions 3 Level Version (29)		5 items	x	x	x	x
	TOP, Trauma Outcome Profile (30)	anxiousness	4 items	x	x	x	x
		depression	4 items	x	x	x	x
		posttraumatic stress disorder	4 items	x	x	x	x
		pain	15 items	x	x	x	x
		body functions	15 items	x	x	x	x
		social interaction/ financial problems	4 items		x	x	x
		activity of daily living (ADL)	4 items		x	x	x
		mental function	4 items		x	x	x
		body image	1 item		x	x	x
		overall satisfaction	1 item		x	x	x
	Short-Form-Health-Survey-12 (31)		12 items		x	x	x
	*self-constructed questions*		Likert scale: self-appraisal of health and wellness	x	x	x	x
			sleep quality		x	x	x
			spirituality		x	x	
			sexual life				x
circumstances of accident	*self-constructed questions*		accident framework	x			
			accident recall	x			
			presence of accident opponent	x			
			ongoing claims		x	x	x
			subjective or objective fault	x			
perception of acute care treatment	*self-constructed questions*		evaluation of preclinical treatment	x			
			evaluation of emergency department treatment	x			
			Information level regarding treatment	x			
			Likert scale: pain treatment	x			
return to work	*self-constructed questions*		health insurance		x	x	
			ongoing work incapacity		x	x	x
			occupational rehabilitation		x	x	x
			change of income or work hours		x	x	x
			occupational redeployment		x	x	x
			current occupation		x	x	x
			commute		x	x	x
			support by employer			x	x
rehabilitation	*self-constructed questions*		current residence		x	x	x
			information level of treatment		x	x	x
			rehabilitation outpatient or inpatient		x	x	x
			direct transfer to rehabilitation		x	x	x
			length of rehabilitation		x	x	x
			evaluation of exercises, tailored rehabilitation programm		x	x	x
			participation opportunity of excercises		x	x	x
			subjective recovery after rehabilitation		x	x	x
			evaluation of discharge- management		x	x	
			ongoing physiotherapy after rehabilitation		x	x	x
			evaluation of rehabilitation manager		x	x	x
secondary gain of trauma	FPTM – Questionnaire for assessing psychotherapy motivation (32)	symptom-focused attention from others	6 items			x	
body shame	*self-constructed questions*		7 items			x	
post hospital treatment	*self-constructed questions*		evaluation of discharge management		x	x	x
			helplessness after discharge		x	x	x
			information level at time of discharge		x	x	
			ongoing physiotherapy after hospital treatment		x	x	x
			Speciality/profession of treating physician		x	x	x
			evaluation of treatment coordination by physician		x	x	x
			Likert-scale: evaluation of pain therapy		x	x	x
			ongoing psychological, psychiatric or psychotherapeutic treatment		x	x	x
			access to post hospital paramedical treatment		x	x	x
			quality of contact with authorities regarding trauma consequences		x	x	x
			evaluation of hospital treatment			x	
			evaluation of post-hospital treatment			x	
			helplessness after discharge from hospital		x	x	x
medical courses of trauma	*self-constructed questions*		complications during the healing processFormularbeginnFormularende		x	x	x
			further operations after discharge from hospital		x	x	x
			ongoing treatment regarding accident				x
personality	Big Five Factor Model (33)		10 items			x	
PTSD – posttraumatic stress dissorder	International trauma questionnaire (34)		9 items				x
treatment evaluation	*self-constructed questions*		overall evaluation of treatment				x
			evaluation of general life situation after trauma				x
			change on outlook on life after trauma				x
			problems occurring during post hospital care				x
							

**Fig 1:**
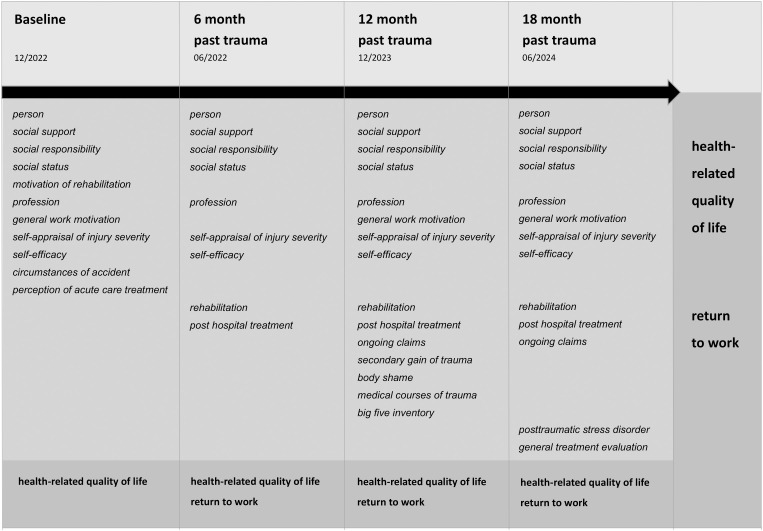
Overview of measurement time, predictors and outcome parameters of LeAf Trauma.

**Fig 2:**
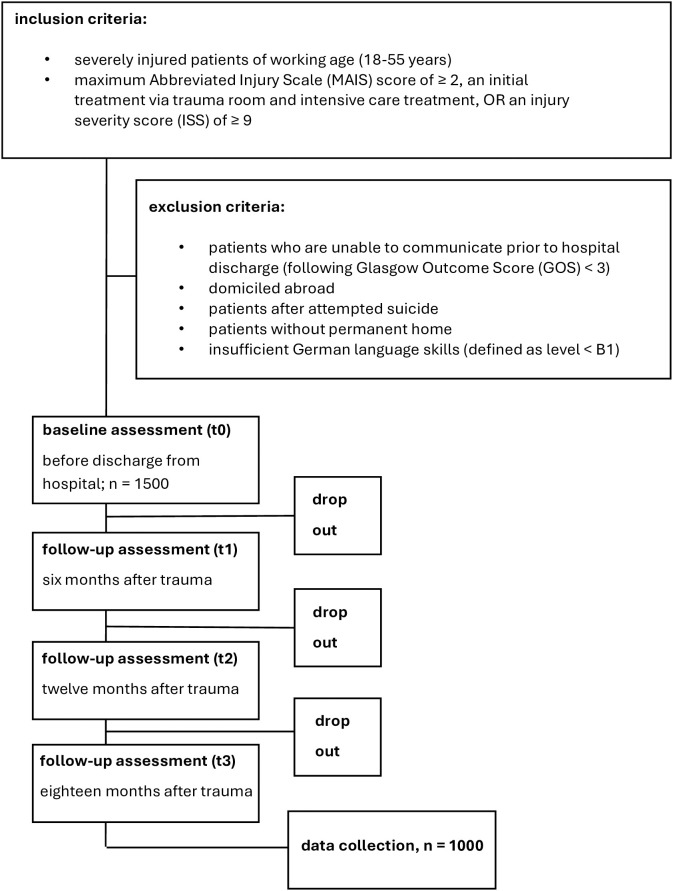
Inclusion- and exclusion criteria with expected drop-out rates.

Light grey: measurement time points, grey: predictor, dark grey: outcome.

In the Patient recruitment and data collection subsection of Methods, there is an error in the second and seventh sentence of the second paragraph. The correct second sentence is: These trauma centers receive a compensation of maximal 660 € per patient staggered according to the number of successfully documented follow- ups.

The correct seventh sentence is: On special request, if online access is not available for the patient, paper-pencil documents are sent.

In the Inclusion and exclusion criteria subsection of the Methods, there is an error in the first, third and fourth sentences of the paragraph.

The correct first sentence is: For the prospective study, patients are recruited from 12/2022–11/2024.

The correct third and fourth sentences are: Exclusion criteria are patients who are not able to communicate after discharge from hospital, following Glasgow Outcome Score (GOS) *<*3 [15], domiciled abroad, persons after attempted suicide, patients without permanent home or no sufficient German language skills (defined as level ≥ B1, European Reference). In 2023, the inclusion criteria have been amended to foster inclusion rates.

In the Sample size analysis subsection of the Methods, there is an error in the second and third sentences of the first paragraph. The correct sentences are: Therefore, the collective must be large enough to examine socioeconomic and psychosocial factors in addition to patient- and injury-specific subgroup analyses (e.g., injury of lower extremities). With a total sample size of n = 1000 evaluable cases (with complete follow-up), a prevalence of 10% of an outcome criterion could be given with an accuracy of +/-2% (95% confidence interval), a prevalence of 20% with +/- 2.5%, and a prevalence of 50% with +/- 3%.

In the Sample size analysis subsection of the Methods, there is an error in the third sentence of the second paragraph. The correct sentence is: A response rate of about 50–66% is assumed [16], of which it is expected that 25–30% will not complete all follow-up assessments.

In the Statistical analysis subsection of the Methods, there is an error in the first sentence of the first paragraph. The correct sentence is: Data will be exported from the AUC registry platform [17] and LimeSurvey [14] to IBM SPSS statistics for analysis.

In the Discussion, there is an error in the seventh sentence. The correct sentence is: Through the retrospective study arm initial consolidations of ICD-10 coding into useful trauma constellations is made.

There are errors in the Author Contributions. The correct contributions are:

**Conceptualization:** Dan Bieler, Christine Hoefer, Rolf Lefering

**Funding acquisition:** Christine Hoefer, Dan Bieler

**Investigation:** Katharina Fetz, Anne Neubert

**Methodology:** Katharina Fetz, Gina Grimaldi, Dan Bieler, Anne Neubert, Jean-Jacques Glaesener, Rolf Lefering

**Project administration:** Christine Hoefer, Elisabeth Schwojer, Stefanie Bartha

**Supervision:** Dan Bieler, Carina Jaekel, Christine Hoefer

**Visualization:** Gina Grimaldi

**Writing – original draft:** Katharina Fetz, Gina Grimaldi, Anne Neubert

**Writing – review & editing:** Katharina Fetz, Gina Grimaldi, Dan Bieler, Anne Neubert, Carina Jaekel, Christine Hoefer, Elisabeth Schwojer, Stefanie Bartha, Jean-Jacques Glaesener, Lars Becker, Lisa Wienhoefer, Rolf Lefering

There is an error in references 17 and 20. The correct references are:

17. AUC registry platform. https://www.traumaregister-dgu.de/index.php

20. Deutsches Institut für Medizinische Dokumentation und Information (DIMDI) im Auftrag des Bundesministeriums für Gesundheit (BMG) unter Beteiligung der Arbeitsgruppe ICD des Kuratoriums für Fragen der Klassifikation im Gesundheitswesen (KKG) (Hrsg.) (2017). ICD-10-GM Version 2017. Systematisches Verzeichnis Internationale statistische Klassifikation der Krankheiten und verwandter Gesundheitsprobleme (10. Revision, German Modification). Unter: http://www.dimdi.de/static/de/klassi/icd-10-gm/kodesuche/onlinefassungen/htmlgm2017/block-a92-a99.htm

The publisher apologizes for the errors.
