# Supplementary material for: LeAf Trauma- an intersectoral prospective multicenter study assessing quality of life and return to work after majortrauma–study protocol
Source: PLoS One. 2024 Nov 13;19(11):e0312320. doi: 10.1371/journal.pone.0312320 (PMC11560036; doi:10.1371/journal.pone.0312320)
Supplement: S1 File — (PDF) [file pone.0312320.s001.pdf]

SPIRIT

STANDARD PROTOCOL ITEMS: RECOMMENDATIONS FOR INTERVENTIONAL TRIALS

Table 1 SPIRIT Schedule

| | STUDY PERIOD | | | | | | |
|---|---|---|---|---|---|---|---|
| | **Enrolment** | **Allocation *** | **Measurement timepoints** | | | | **completion of data collection** |
| **TIMEPOINT** | ***10/2022*** | **-** | ***12/2022*** | ***06/2023*** | ***12/2023*** | ***06/2024*** | ***02/2026*** |
| **ENROLMENT:** | | | | | | | |
| **Data security concept** | X | | | | | | |
| **Definition of eligibility criteria** | X | | | | | | |
| **Study registration** | X | | | | | | |
| **Funding Approval** | X | | | | | | |
| **Ethics approval** | X | | | | | | |
| **Cooperation with Study centers** | X | | | | | | |
| **Informed consent** | | | X | | | | |
| **ASSESSMENTS**:** | | | | | | | |

| | | | | | | | |
|---|---|---|---|---|---|---|---|
| **medical details** | | | X | | | | |
| **personal information** | | | X | X | X | X | |
| **social status** | | | X | X | X | X | |
| **motivation of rehabilitation** | | | X | | | | |
| **profession** | | | X | | | | |
| **general work motivation** | | | X | | X | X | |
| **self-efficacy and resilience** | | | X | X | X | X | |
| **circumstances of accident** | | | X | X | X | X | |
| **secondary gain of trauma** | | | | | X | | |
| **body shame** | | | | | X | | |
| **medical courses of trauma** | | | | X | X | X | |
| **personality** | | | | | X | | |
| **posttraumatic stress dissorder** | | | | | | X | |
| **PREM*** ** | | | X | X | X | X | |
| **PROM*** ** | | | | X | X | X | |

| | | | | | | | |
|---|---|---|---|---|---|---|---|
| **EVALUATION:** | | | | | | | X |
| **subgroup analysis** | | | | | | | X |
| **recommendations regarding major-trauma patients** | | | | | | | X |

SPIRIT 2013 Checklist: Recommended items to address in a clinical trial protocol and related documents*

| **Section/item** | **ItemNo** | **Description** | **Section, page** |
|---|---|---|---|
| **Administrative information** | | | |
| Title | 1 | Descriptive title identifying the study design, population, interventions, and, if applicable, trial acronym | See title. |
| Trial registration | 2a | Trial identifier and registry name. If not yet registered, name of intended registry | German Clinical Trials Register (DRKS): DRKS00028841 |
| | 2b | All items from the World Health Organization Trial Registration Data Set | See below: WHO Trial Registration Data Set |
| Protocol version | 3 | Date and version identifier | See study protocol. |
| Funding | 4 | Sources and types of financial, material, and other support | Funding Code: 01VSF21033<br>Funding Period: 01.04.2022 – 31.03.2026 |

| | | | |
|---|---|---|---|
| Roles and responsibilities | 5a | Names, affiliations, and roles of protocol contributors | See manuscript: Author's Contributions, page 15 |
| | 5b | Name and contact information for the trial sponsor | Innovationsausschuss beim Gemeinsamen Bundesausschuss<br>Gutenbergstraße 13<br>10587 Berlin<br>Germany<br>E-Mail: info@if.g-ba.de<br><br>AUC-Akademie der Unfallchirurgie GmbH<br>Dr.-Ing. Christine Höfer<br>Emil-Riedel-Str. 5<br>80538 München<br>leaf-trauma@auc-online.de<br>www.auc-online.de |
| | 5c | Role of study sponsor and funders, if any, in study design; collection, management, analysis, and interpretation of data; writing of the report; and the decision to submit the report for publication, including whether they will have ultimate authority over any of these activities | AUC is responsible for providing eCRF, management and data quality check and in cooperation with partner IFOM responsible for interpretation of the data and writing of the report. AUC as lead of the consortium has the authority over the aforementioned activities.<br>Changes to the study protocol must be reported to and approved by the funding organisation Innovationsfond am G-BA. |

| | | | |
|---|---|---|---|
| | 5d | Composition, roles, and responsibilities of the coordinating centre, steering committee, endpoint adjudication committee, data management team, and other individuals or groups overseeing the trial, if applicable (see Item 21a for data monitoring committee) | The consortium partners built the steering committee of the study. They have to address their respective working packages within the study. Changes to the planned procedure are discussed and decided by the steering committee. |
| **Introduction** | | | |
| Background and rationale | 6a | Description of research question and justification for undertaking the trial, including summary of relevant studies (published and unpublished) examining benefits and harms for each intervention | Introduction section, page 2 |
| | 6b | Explanation for choice of comparators | |
| Objectives | 7 | Specific objectives or hypotheses | Introduction, page 2<br>Methods, page 4 |
| Trial design | 8 | Description of trial design including type of trial (eg, parallel group, crossover, factorial, single group), allocation ratio, and framework (eg, superiority, equivalence, noninferiority, exploratory) | n.a.<br>Our study is a prospective cohort study. |
| **Methods: Participants, interventions, and outcomes** | | | |

| | | | |
|---|---|---|---|
| Study setting | 9 | Description of study settings (eg, community clinic, academic hospital) and list of countries where data will be collected. Reference to where list of study sites can be obtained | Methods, page 4<br>All study clinics are listed in https://www.leaf-trauma.de/kliniken |
| Eligibility criteria | 10 | Inclusion and exclusion criteria for participants. If applicable, eligibility criteria for study centres and individuals who will perform the interventions (eg, surgeons, psychotherapists) | Methods section, page 4 |
| Interventions | 11a | Interventions for each group with sufficient detail to allow replication, including how and when they will be administered | Not applicable, there is no intervention. |
| | 11b | Criteria for discontinuing or modifying allocated interventions for a given trial participant (eg, drug dose change in response to harms, participant request, or improving/worsening disease) | Not applicable, there is no intervention. |
| | 11c | Strategies to improve adherence to intervention protocols, and any procedures for monitoring adherence (eg, drug tablet return, laboratory tests) | Not applicable, there is no intervention. |
| | 11d | Relevant concomitant care and interventions that are permitted or prohibited during the trial | Not applicable, there is no intervention. |

| | | | |
|---|---|---|---|
| Outcomes | 12 | Primary, secondary, and other outcomes, including the specific measurement variable (eg, systolic blood pressure), analysis metric (eg, change from baseline, final value, time to event), method of aggregation (eg, median, proportion), and time point for each outcome. Explanation of the clinical relevance of chosen efficacy and harm outcomes is strongly recommended | Primary outcomes:<br>• Return to work<br>• Health related quality of life<br><br>Secondary outcomes:<br>• PROMs<br>• PREMS<br><br>See Table 1 and Table 2 |
| Participant timeline | 13 | Time schedule of enrolment, interventions (including any run-ins and washouts), assessments, and visits for participants. A schematic diagram is highly recommended (see Figure) | We added the measures to the spirit flowchart. For more detailed version see Fig 2 |
| Sample size | 14 | Estimated number of participants needed to achieve study objectives and how it was determined, including clinical and statistical assumptions supporting any sample size calculations | Statistics section, sample size analysis page 9 |
| Recruitment | 15 | Strategies for achieving adequate participant enrolment to reach target sample size | Statistics section, page 9 |
| **Methods: Assignment of interventions (for controlled trials)** | | | **Not applicable – This study is no controlled trial** |
| Allocation: | | | n.a. |

| | | | |
|---|---|---|---|
| Sequence generation | 16a | Method of generating the allocation sequence (eg, computer-generated random numbers), and list of any factors for stratification. To reduce predictability of a random sequence, details of any planned restriction (eg, blocking) should be provided in a separate document that is unavailable to those who enrol participants or assign interventions | n.a. |
| Allocation concealment mechanism | 16b | Mechanism of implementing the allocation sequence (eg, central telephone; sequentially numbered, opaque, sealed envelopes), describing any steps to conceal the sequence until interventions are assigned | n.a. |
| Implementation | 16c | Who will generate the allocation sequence, who will enrol participants, and who will assign participants to interventions | n.a. |
| Blinding (masking) | 17a | Who will be blinded after assignment to interventions (eg, trial participants, care providers, outcome assessors, data analysts), and how | Not applicable, there is no intervention and no blinding. |
| | 17b | If blinded, circumstances under which unblinding is permissible, and procedure for revealing a participant's allocated intervention during the trial | n.a. |
| **Methods: Data collection, management, and analysis** | | | |

| | | | |
|---|---|---|---|
| Data collection methods | 18a | Plans for assessment and collection of outcome, baseline, and other trial data, including any related processes to promote data quality (eg, duplicate measurements, training of assessors) and a description of study instruments (eg, questionnaires, laboratory tests) along with their reliability and validity, if known. Reference to where data collection forms can be found, if not in the protocol | Methods section, page 8 |
| | 18b | Plans to promote participant retention and complete follow-up, including list of any outcome data to be collected for participants who discontinue or deviate from intervention protocols | Patients received extensive information material in the form of brochures and flyers when they joined the study, which also provided detailed information about the follow-up interviews. Follow Up reminder are dispatched several times to the study clinics by AUC if data are missing in the eCRF. Monthly reports are submitted to the study clinics on their performance including a ranking list and follow up rate. As the study clinics are responsible for contacting the patients, their commitment is promoted by staggered reimbursements for each follow up survey. |

| | | | |
|---|---|---|---|
| Data management | 19 | Plans for data entry, coding, security, and storage, including any related processes to promote data quality (eg, double data entry; range checks for data values). Reference to where details of data management procedures can be found, if not in the protocol | Data quality is systematically checked by functionalities of the eCRF Software using mandatory fields, extensive plausibility checks, limit value checks and a detailed query management and feedback to the study clinics. |
| Statistical methods | 20a | Statistical methods for analysing primary and secondary outcomes. Reference to where other details of the statistical analysis plan can be found, if not in the protocol | Statistics section, page 10 |
| | 20b | Methods for any additional analyses (eg, subgroup and adjusted analyses) | Statistics section, page 10 |
| | 20c | Definition of analysis population relating to protocol non-adherence (eg, as randomised analysis), and any statistical methods to handle missing data (eg, multiple imputation) | Statistics section, page 10 |
| **Methods: Monitoring** | | | |
| Data monitoring | 21a | Composition of data monitoring committee (DMC); summary of its role and reporting structure; statement of whether it is independent from the sponsor and competing interests; and reference to where further details about its charter can be found, if not in the protocol. Alternatively, an explanation of why a DMC is not needed | No need for a DMC due to minimal risks / no safety concerns (non-interventional cohort study)<br><br>Data monitoring is performed by AUC. |

| | | | |
|---|---|---|---|
| | 21b | Description of any interim analyses and stopping guidelines, including who will have access to these interim results and make the final decision to terminate the trial | n.a. |
| Harms | 22 | Plans for collecting, assessing, reporting, and managing solicited and spontaneously reported adverse events and other unintended effects of trial interventions or trial conduct | n.a. |
| Auditing | 23 | Frequency and procedures for auditing trial conduct, if any, and whether the process will be independent from investigators and the sponsor | n.a. |
| **Ethics and dissemination** | | | |
| Research ethics approval | 24 | Plans for seeking research ethics committee/institutional review board (REC/IRB) approval | Ethics Committee at the Faculty of Medicine<br>Department of Orthopedics and Trauma Surgery, UKD<br>Prof. Dr. med. Joachim Windolf<br>Moorenstr 5<br>40225 Düsseldorf<br><br>Study No.: 2022-2029-other research first voting<br><br>17.10.2022 |

| | | | |
|---|---|---|---|
| Protocol amendments | 25 | Plans for communicating important protocol modifications (eg, changes to eligibility criteria, outcomes, analyses) to relevant parties (eg, investigators, REC/IRBs, trial participants, trial registries, journals, regulators) | changes to eligibility criteria were performed in 08/2023 |
| Consent or assent | 26a | Who will obtain informed consent or assent from potential trial participants or authorised surrogates, and how (see Item 32) | Informed consent is obtained by the local study center (for a complete list of study centers see https://www.leaf-trauma.de/kliniken)<br><br>Qualifying patients are personally informed about the study by the study staff during their hospital stay. They are given written information material and, after an appropriate period of reflection, consent is obtained. Patients that are not participating the study are listed in the individual study clinic´s consecutive list of qualifying patients with the comment: no consent. |
| | 26b | Additional consent provisions for collection and use of participant data and biological specimens in ancillary studies, if applicable | Patients are asked for their consent for further scientific use of the data in the context of major trauma as well as for participation in the TraumaRegister DGU® www.traumaregister-dgu.de |

| | | | |
|---|---|---|---|
| Confidentiality | 27 | How personal information about potential and enrolled participants will be collected, shared, and maintained in order to protect confidentiality before, during, and after the trial | see “data Management and Data Protection”, study protocol page 13 |
| Declaration of interests | 28 | Financial and other competing interests for principal investigators for the overall trial and each study site | The authors declare no competing interest. |
| Access to data | 29 | Statement of who will have access to the final trial dataset, and disclosure of contractual agreements that limit such access for investigators | Consortial partners as listed as LeAf Trauma study group.<br><br>The final trial dataset is accessed only by AUC and the partner IFOM. This is defined by the agreed working packages and the project´s data protection concept. Research by third parties with disclosure of raw data is not planned and not the subject of the consent obtained. Scientific reuse however is possible in cooperation and with sole access to the raw data by the AUC. |
| Ancillary and post-trial care | 30 | Provisions, if any, for ancillary and post-trial care, and for compensation to those who suffer harm from trial participation | n.a. |

| | | | |
|---|---|---|---|
| Dissemination policy | 31a | Plans for investigators and sponsor to communicate trial results to participants, healthcare professionals, the public, and other relevant groups (eg, via publication, reporting in results databases, or other data sharing arrangements), including any publication restrictions | Trial results will be communicated via publication. |
| | 31b | Authorship eligibility guidelines and any intended use of professional writers | n.a. |
| | 31c | Plans, if any, for granting public access to the full protocol, participant-level dataset, and statistical code | See 29. Participant-level dataset will not be available for open access. Therefore it is not planned to grant public access to full protocol or statistical code. |
| **Appendices** | | | |
| Informed consent materials | 32 | Model consent form and other related documentation given to participants and authorised surrogates | See “patient information and patient consent form”. |
| Biological specimens | 33 | Plans for collection, laboratory evaluation, and storage of biological specimens for genetic or molecular analysis in the current trial and for future use in ancillary studies, if applicable | Not applicable |

*It is strongly recommended that this checklist be read in conjunction with the SPIRIT 2013 Explanation & Elaboration for important clarification on the items. Amendments to the protocol should be tracked and dated. The SPIRIT checklist is copyrighted by the SPIRIT Group under the Creative Commons “Attribution-NonCommercial-NoDerivs 3.0 Unported” license.
