# Supplementary material for: LeAf Trauma- an intersectoral prospective multicenter study assessing quality of life and return to work after majortrauma–study protocol
Source: PLoS One. 2024 Nov 13;19(11):e0312320. doi: 10.1371/journal.pone.0312320 (PMC11560036; doi:10.1371/journal.pone.0312320)
Supplement: S2 File — (PDF) [file pone.0312320.s002.pdf]

Nr.	Study clinic	Issuing ethics committee	Positive ethics vote	Date
1	Universitätsmedizin Rostock	Ethikkommission an der Universitätsmedizin Rostock	Written confirmation	21.09.22
2	Universitätsklinikum Düsseldorf	Ethikkommission an der Medizinischen Fakultät der Heinrich-Heine-Universität Düsseldorf	Written confirmation	17.10.22
3	Universitätsklinikum Schleswig-Holstein, Campus Lübeck	Ethik-Kommission an der Universität zu Lübeck	Written confirmation	25.10.22
4	Universitätsklinikum Regensburg	Ethikkommission bei der Universität Regensburg	Written confirmation	25.10.22
5	BG Klinikum Hamburg	Ethik-Kommission der Ärztekammer Hamburg	Written confirmation	01.11.22
6	Medizinische Hochschule Hannover, Unfallchirurgische Klinik	Ethikkommission an der Medizinischen Hochschule Hannover	Written confirmation	03.11.22
7	Uniklinik RWTH Aachen	Ethik-Kommission an der medizinischen Fakultät der RWTH Aachen	Written confirmation	04.11.22
8	Klinikum Ernst von Bergmann, Potsdam	Ethikkommission der Landesärztekammer Brandenburg	Written confirmation	07.11.22
14	Klinikum Kassel	Ethik-Kommission bei der Landesärztekammer Hessen	Written confirmation	11.11.22
15	Universitätsmedizin Mannheim	Ethik-Kommission II der Universität Heidelberg	Written confirmation	17.11.22
18	Bergmannsheil und Kinderklinik Buer	Ethik-Kommission der Ärztekammer Westfalen-Lippe und der Westfälischen Wilhelms-Universität	Written confirmation	21.11.22
19	Diakonie Klinikum, Jung-Stilling Krankenhaus	Ethik-Kommission der Ärztekammer Westfalen-Lippe und der Westfälischen Wilhelms-Universität	Written confirmation	21.11.22
20	Klinikum Dortmund	Ethik-Kommission der Ärztekammer Westfalen-Lippe und der Westfälischen Wilhelms-Universität	Written confirmation	21.11.22
21	Evangelisches Klinikum Bethel	Ethik-Kommission der Ärztekammer Westfalen-Lippe und der Westfälischen Wilhelms-Universität	Written confirmation	21.11.22
22	Universitätsklinikum Gießen und Marburg, Marburg	Ethikkommission des FB Medizin der Philipps-Universität Marburg	Written confirmation	23.11.22

26	Städtisches Klinikum Dresden	Ethikkommission der Sächsischen Landesärztekammer	Written confirmation	25.11.22
27	Universitätsklinikum Freiburg	Ethik-Kommission an der Albert-Ludwigs-Universität Freiburg	Written confirmation	29.11.22
23	Ortenau Klinikum Offenburg-Kehl, Unfallklinik Offenburg	Ethik-Kommission der Landesärztekammer Baden-Württemberg	Written confirmation	30.11.22
24	Städtisches Klinikum Karlsruhe	Ethik-Kommission der Landesärztekammer Baden-Württemberg	Written confirmation	30.11.22
25	Hegau-Bodensee-Klinikum Singen	Ethik-Kommission der Landesärztekammer Baden-Württemberg	Written confirmation	30.11.22
28	Universitätsklinikum Gießen und Marburg, Gießen	Ethik-Kommission des FB Medizin der Justus-Liebig Universität Gießen	Written confirmation	09.12.22
29	BG Universitätsklinikum Bergmannsheil	Ethik-Kommission der Medizinischen Fakultät der Ruhr Universität Bochum	Written confirmation	13.12.22
30	Klinikum Köln-Merheim	Ethik-Kommission der Universität Witten / Herdecke	Written confirmation	15.12.22
31	Universitätsklinikum Augsburg	Ethikkommission bei der Ludwig-Maximilians-Universität München	Written confirmation	04.01.23
33	LMU Klinikum Großhadern	Ethikkommission bei der Ludwig-Maximilians-Universität München	Written confirmation	10.01.23
34	Uniklinikum Magdeburg	Ethikkommission der Otto-von-Guericke-Universität Magdeburg	Written confirmation	16.12.22
35	BG Unfallklinik Frankfurt am Main	Ethik-Kommission bei der Landesärztekammer Hessen	Written confirmation	17.01.23
36	Asklepios Klinik St. Georg	Ethik-Kommission der Ärztekammer Hamburg	Written confirmation	24.01.23
37	BG Klinik Tübingen	Ethik-Kommission Med. Fakultät der Eberhard-Karls-Universität/ Universitätsklinikum Tübingen	Written confirmation	22.01.23
38	Universitätsklinikum Leipzig	Ethik-Kommission an der Medizinischen Fakultät der Universität Leipzig	Written confirmation	22.12.22

39	Universitätsklinikum Carl Gustav Carus Dresden	Ethikkommission an der TU Dresden	Written confirmation	26.01.23
40	BG Klinikum Ludwigshafen	Ethikkommission der Landesärztekammer Rhenland-Pfalz	Written confirmation	07.02.23
41	Krankenhaus der Barmherzigen Brüder Trier	Ethikkommission der Landesärztekammer Rhenland-Pfalz	Written confirmation	07.02.23
42	Westpfalz-Klinikum	Ethikkommission der Landesärztekammer Rhenland-Pfalz	Written confirmation	07.02.23
43	Universitätsklinikum Erlangen	Ethik-Kommission der Friedrich-Alexander-Universität Erlangen	Written confirmation	22.02.23
44	Klinikum Magdeburg	Ethikkommission der Ärztekammer Sachsen-Anhalt	Written confirmation	23.02.23
45	BG Klinikum Duisburg	Ethik-Kommission der Ärztekammer Nordrhein	Written confirmation	09.03.23
47	Uniklinikum Göttingen	Ethik-Kommission der Universitätsmedizin Göttingen	Written confirmation	21.03.23
48	Helios Universitätsklinikum Wuppertal	Ethik-Kommission der Ärztekammer Nordrhein	Written confirmation	20.04.23
49	BG Klinikum Bergmannstrost Halle	Ethikkommission der Ärztekammer Sachsen-Anhalt	Written confirmation	19.04.23
50	Medizinische Universität Graz	Ethikkommission der Medizinische Universität Graz	Written confirmation	02.05.23
51	Bundeswehrzentralkrankenhaus Koblenz	Ethikkommission der Landesärztekammer Rhenland-Pfalz	Written confirmation	26.06.23
52	Universitätsmedizin Essen	Ethikkommission der Medizinischen Fakultät Duisburg-Essen	Written confirmation	10.07.23

**No separate ethics vote required**				
16	Vivantes Klinikum Friedrichshain	Ethik-Kommission der Ärztekammer Berlin	Written confirmation	18.11.22
17	BG Klinikum Unfallkrankenhaus Berlin	Ethik-Kommission der Ärztekammer Berlin	Written confirmation	18.11.22
9	Klinikum Passau	Ethik-Kommission der Bayerischen Landesärztekammer	Written confirmation	08.11.22

10	Krankenhaus Barmherzige Brüder Regensburg	Ethik-Kommission der Bayerischen Landesärztekammer	Written confirmation	08.11.22
11	BG Unfallklinik Murnau	Ethik-Kommission der Bayerischen Landesärztekammer	Written confirmation	08.11.22
