# Supplementary material for: LeAf Trauma- an intersectoral prospective multicenter study assessing quality of life and return to work after majortrauma–study protocol
Source: PLoS One. 2024 Nov 13;19(11):e0312320. doi: 10.1371/journal.pone.0312320 (PMC11560036; doi:10.1371/journal.pone.0312320)
Supplement: S3 File — (PDF) [file pone.0312320.s003.pdf]

S3 - Patient Information and Patient Consent Form

Patient Information Regarding Participation in the LeAf Trauma Study

Dear Patient,

Approximately 30,000 people in Germany suffer a severe trauma annually. Unfortunately, you are also affected.

The team treating you, consisting of doctors, nursing staff, and physiotherapists, is doing everything possible to minimize the consequences for you. After your discharge from the hospital, you will face a longer phase of rehabilitation, as the overall goal of the treatment is to restore your quality of life and independence as best as possible.

Given the significant individual impact on those affected, the German Society for Trauma Surgery (Deutsche Gesellschaft für Unfallchirurgie e.V.) and teams of doctors and scientists are continuously striving to research and improve treatment outcomes.

One such research project, supported by the doctors and nursing staff at your treating hospital, is the LeAf Trauma Project, led by the AUC Academy of Trauma Surgery. This project is financed by the Innovation Fund of the Joint Federal Committee until September 30, 2025.

Goal of the LeAf Trauma Study

The aim is to learn more about your treatment course, recovery, and return to work after discharge from the initial acute hospital stay. Insights gained will be used to derive measures to improve the quality of life for severely injured patients and to promote a quicker return to independence.

We would like to ask for your support by participating in the LeAf Trauma Study. Your personal participation is simple and requires minimal effort from you. Additionally, your participation poses no health risks, and you will not be subjected to any new treatment methods.

During your hospital treatment and after inclusion in the study, you will be visited by clinic staff specifically trained for this study, and medical information related to your injury and treatment will be collected. You will also be asked about your quality of life before the accident and your occupation. After your discharge from the hospital, we will contact you three times at 6, 12, and 18 months, either by phone or in writing, to inquire about your current personal situation, well-being, and further treatment or rehabilitation measures. The details of the follow-up surveys are included in your study documents. You can also view them at any time on the project website (www.leaf-trauma.de) or inquire from the study management at the AUC Academy of Trauma Surgery.

The data collected from you will be pseudonymized, meaning that your personally identifiable data will be replaced by a numerical code, complying with data protection regulations, and transferred to the study registry without your name or address. A list matching the codes to your identity will be maintained only at your treating hospital. The confidentiality of personal data will always be ensured in the publication of study results. Compliance with data

protection requirements is thoroughly outlined in the LeAf Trauma Study's data protection concept.

Your participation in the LeAf Trauma Project makes a significant contribution to researching treatment outcomes and improving care for severely injured patients!

How long is the consent valid?

Your consent for data collection (including follow-up) is valid for the duration of the LeAf Trauma Study. The collected data will be used for study purposes until at most 12 months after the project concludes unless you revoke it earlier.

Duration of Data Retention

The data will be archived for 10 years after the study concludes.

Right to Withdraw

Your consent is voluntary! You can end your participation in the LeAf Trauma Study at any time. If you wish to withdraw, please notify us in writing (letter, fax, or email). Rejoining the study would only be possible if you re-enroll with a new declaration of participation and meet the participation requirements.

In case of withdrawal, your data will be deleted. Data from analyses already conducted cannot be removed retroactively.

To withdraw, please contact: **[Placeholder for the data protection officer at the local study center]**

Whenever data is collected, stored, and transmitted as part of research projects involving patient data, there is a residual risk that additional information, such as from the internet or social networks, could be used to trace back to your identity. This is especially true if you publish genetic or other health data, such as for genealogy research, on the internet. Should your data, despite extensive technical and organizational protective measures, fall into unauthorized hands and a link to your identity be made despite the absence of name information, the data could potentially be used in a discriminatory or otherwise harmful manner to you and possibly close relatives.

The local study center is responsible for collecting and passing on your patient data: Placeholder for the local study center (respective hospital) The data protection officer at your local study center is: **[Placeholder for the data protection officer at the local study center]**

The supervisory authority responsible for your local study center is: **[Placeholder for the data protection officer at the local study center]**

The responsible entity for subsequent data processing for research purposes is the study management and consortium leadership: AUC-Academy of Trauma Surgery Emil-Riedel-Straße 5 80538 Munich Telephone: 089 540481 100 together with the other data protection responsible entities of the LeAf Trauma Consortium. These can be found in the table below.

The data protection officer of the study management can be reached at: AUC-Academy of Trauma Surgery Emil-Riedel-Straße 5 80538 Munich Email: datenschutz@auc-online.de

Further Information and Rights The legal basis for data processing is your consent according to Article 9 Paragraph 2 a) and Article 6 Paragraph 1 a) of the European General Data Protection Regulation (GDPR). You have the option to file a complaint with any data protection supervisory authority. A list of supervisory authorities in Germany can be found at: https://www.bfdi.bund.de/DE/Infothek/Anschriften_Links/anschriften_links-node.html

The supervisory authority responsible for the project management is the Bavarian State Office for Data Protection Supervision: Bavarian State Office for Data Protection Supervision (BayLDA) Promenade 18 91522 Ansbach https://www.lda.bayern.de

Additionally, you have the right to request information about the personal data processed by us and related information, including a free copy upon request (Art. 15 GDPR). In the event that personal data about you is not (or is no longer) accurate or incomplete, you can request the correction and, if necessary, the completion of this data (Art. 16 GDPR). If the legal requirements are met, you can request the deletion of your personal data (Art. 17 GDPR) or the restriction of the processing of this data (Art. 18 GDPR).

Furthermore, you have the right to receive data you have provided in a standardized electronic format or to have it transmitted to a designated third party (Right to Data Portability according to Art. 20 GDPR).

Patient Information on the Use of Patient Data for Medical Research Purposes

Dear Patient,

You are currently receiving medical treatment for a severe trauma and are being asked to participate in the LeAf Trauma study. As part of your study participation, patient data will be collected from you. This patient data can be of significant value for medical research beyond the LeAf Trauma study. Medical research is necessary to continuously improve the early detection, treatment, and prevention of diseases; insights gained from your patient data can potentially contribute greatly to this. Therefore, we ask for your permission to use your patient data, collected as part of the LeAf Trauma study, for medical research purposes. Your patient data will be stored and researched by the AUC Academy of Trauma Surgery. Your consent is voluntary. If you do not wish to participate or if you wish to withdraw your consent later, there will be no disadvantages for you. If you are not fully satisfied with the type and long-term use described below or if your questions have not been answered satisfactorily, you should not give your consent.

1. Collection, Processing, and Scientific Use of Your Patient Data

1.1 What Are Our Objectives?

Your patient data will be made available for medical research. Medical research aims solely to improve the detection, treatment, and prevention of diseases. Your patient data will not be

used for the development of biological weapons or discriminatory research objectives. Likewise, this research does not aim to diagnose you or influence your specific treatment.

Your patient data is intended to be used for many different medical research purposes for the broad benefit of the general public. At this time, not all future medical research content can be described; it may relate to entire fields of diseases (e.g., treatment of severely injured patients, rehabilitation of severely injured patients, and treatment outcomes for severely injured patients) as well as to individual diseases or injury consequences that are currently unknown. Therefore, your patient data may be used for research questions that we cannot yet foresee. Your patient data, collected as part of the LeAf Trauma study, will be stored for 10 years from the time of your consent, unless you revoke it earlier.

1.2 How Will Your Patient Data Be Used Scientifically?

Your patient data may be used by the AUC Academy of Trauma Surgery for specified and approved research purposes, which are applied for and approved by an expert panel of the German Society for Trauma Surgery (Review Board). Your patient data will be used solely for scientific purposes; it will not be sold or passed on. However, the AUC Academy of Trauma Surgery may charge an appropriate fee for the research assignments on the data from the respective applicants. The permissibility of each individual research project using your patient data will be reviewed in advance by an independent ethics committee and requires their positive evaluation. Scientific publications of results will be done exclusively in an anonymized form, meaning that no conclusions can be drawn about your identity.

Definition "Anonymization" Anonymization involves modifying your data so that it can no longer be associated with you or only with a disproportionately large technical effort.

1.3 Who Has Access to Your Patient Data and How Is It Protected?

Your directly identifying data (name, date of birth, address, etc.) is not included in the LeAf Trauma dataset. Your dataset carries only a combination of characters (coding, pseudonym). This internal identifier and your associated patient data cannot be directly associated with you. The connection between this internal identifier and your directly identifying data is managed only by your treating hospital. Therefore, the patient data provided for medical research cannot be traced back to you or only with disproportionate technical effort. Before your data is used beyond the immediate study purposes of the LeAf Trauma project, a further replacement of the internal identifier by a new combination of characters will take place.

Definition "Coding" When patient data is collected, information such as your name and date of birth is also collected. With such information, it would be easy to identify you personally. However, this information is not transmitted in the LeAf Trauma study but is replaced by a combination of characters by your treating hospital. This way, easy tracing back to you is excluded.

Data that identifies you personally will never be passed on to researchers or other third parties, particularly not to insurance companies or employers. This data is not included in the research dataset.

You will receive information at any time about which studies are being conducted with your data.

1.4 What Risks Are Associated with the Use of Your Patient Data Beyond the LeAf Trauma Project?

Whenever data is collected, stored, and transmitted as part of research projects involving patient data, there is a residual risk that additional information, such as from the internet or social networks, could be used to trace back to your identity. This is especially true if you publish genetic or other health data, such as for genealogy research, on the internet. Should your data, despite extensive technical and organizational protective measures, fall into unauthorized hands and a link to your identity be made despite the absence of name information, the data could potentially be used in a discriminatory or otherwise harmful manner to you and possibly close relatives.

1.5 What Benefit Is There for You Personally?

Personally, you cannot generally expect any immediate advantage or benefit for your health from the scientific use of your patient data. Your current medical treatment will not be affected by your consent. Should the research result in a commercial benefit, e.g., through the development of new drugs or diagnostic procedures, you will not be involved.

1.6 What Benefit Is There for Our Society?

Medical-scientific research projects aim to improve our understanding of disease development and diagnosis, and based on this, to develop improved prevention, care, and treatment approaches.

2. Will There Be Further Contact with You?

Further contact beyond the LeAf Trauma study is not planned.

3. How Long Is Your Consent Valid?

Your consent to the collection of patient data is valid – unless you revoke it earlier (see below) – for the duration of the LeAf Trauma study, i.e., until the completion of the last follow-up survey. Your consent to the processing and use of the data collected thus far remains effective beyond this period (see point 1.1).

4. What Does Your Right to Withdraw Consent Entail?

Your consent is voluntary! You can revoke your consent to the scientific use of your patient data beyond the LeAf Trauma study at any time without giving reasons and without any disadvantageous consequences for you. A revocation always refers only to the future use of your patient data. Data from analyses already carried out cannot be removed retrospectively. In the event of a revocation, your patient data stored on the basis of this consent will be deleted or anonymized, provided this is legally permissible. If deletion is not possible or not feasible with reasonable technical effort, your patient data will be anonymized by deleting the identification code assigned to you. However, the anonymization of your patient data can never completely exclude the later association of information with your identity through other sources. To revoke, please contact your treating hospital. (respective hospital)

5. Further Information and Rights

The legal basis for data processing is your consent (Article 9 Paragraph 2 a and Article 6 Paragraph 1 a of the European General Data Protection Regulation).

Responsible for the collection and transmission of your patient data is: **[Placeholder for contact details of the local study center]**

The data protection officer responsible at your local study center is:
[Placeholder for the data protection officer at the local study center]

The supervisory authority responsible for your local study center is:
[Placeholder for contact details of the supervisory authority for the local study center]

Responsible for the subsequent processing of the data for research purposes is the study management and consortium leadership: **AUC Academy of Trauma Surgery**
Emil-Riedel-Straße 5
80538 Munich
Phone: 0221 888 239 10
Email: support@auc-online.de

The data protection officer of the study management and consortium leadership can be reached at: **AUC Academy of Trauma Surgery**
Emil-Riedel-Straße 5
80538 Munich
Phone: 089 540 481 100
Email: datenschutz@auc-online.de

You have the option to file a complaint with any data protection supervisory authority. A list of supervisory authorities in Germany can be found at:
https://www.bfdi.bund.de/DE/Infothek/Anschriften_Links/anschriften_links-node.html

The supervisory authority responsible for the AUC Academy of Trauma Surgery is the Bavarian State Office for Data Protection Supervision, Promenade 18, 91522 Ansbach (https://lda.bayern.de).

Additionally, you have the right to request information about the patient data concerning you (including, upon request, a free copy), as well as to request their correction, deletion, or restriction of processing if applicable. You also have the right to receive data you have provided in a standardized electronic format or to have it transmitted to a specified party (right to data portability).
