# Supplementary material for: LeAf Trauma- an intersectoral prospective multicenter study assessing quality of life and return to work after majortrauma–study protocol
Source: PLoS One. 2024 Nov 13;19(11):e0312320. doi: 10.1371/journal.pone.0312320 (PMC11560036; doi:10.1371/journal.pone.0312320)
Supplement: S4 File — (PDF) [file pone.0312320.s004.pdf]

Studienprotokoll

LeAf Trauma - **Le**bensqualität und **A**rbeits**f**ähigkeit nach schwerem **Trauma** **-** Quantitative Studienanteile

Version 1.0 – vom 22.06.2022

Inhaltsverzeichnis

1. Projekttitel 2
2. Zusammenfassung des Projekts 2
3. Verantwortlichkeiten 4
4. Wissenschaftlicher Hintergrund 5
5. Projektziele 7
6. Studienpopulation 7
7. Methodik und Durchführung 11
8. Freiwilligkeit und Rücktritt 18
9. Unerwünschte Wirkungen und Belastungen für Teilnehmer 19
10. Nutzen-Risiko Analyse 19
11. Datenmanagement und Datenschutz 20
Quellen 23

1. Projekttitel

„**LeAf Trauma** - **Le**bensqualität und **A**rbeits**f**ähigkeit nach **Trauma**“

- Quantitative Studienanteile

Hinweis

Dies ist ein Antrag für ein koordiniertes Ethikverfahren. Die hauptverantwortliche Ethikkommission soll hierbei die Ethikkommission der Heinrich-Heine Universität sein. Eine Liste der teilnehmenden Studienkliniken findet sich in den Anhängen.

2. Zusammenfassung des Projekts

2.1 Wissenschaftliche Beschreibung

Das Outcome von schwerverletzten Patienten[1] hängt von multiplen Faktoren ab. Der Behandlungspfad ist gekennzeichnet von Sektorenübertritten (z. B. Rettungsdienst > Krankenhaus > Rehabilitation > ambulante Nachsorge) und interdisziplinärer Behandlung, die verschiedenste Aspekte der Genesung unterstützen. Im Projekt „LeAf Trauma“ sollen die beeinflussbaren Risikofaktoren identifiziert und deren Einfluss quantifiziert werden, um daraus Maßnahmen und Empfehlungen zur Verbesserung der Patientenversorgung und zum Abbau von Hindernissen im Patientenpfad abzuleiten. Die patientenrelevanten Endpunkte sind gesundheitsbezogene Lebensqualität (hrQoL) sowie die Wiederherstellung der Arbeitsfähigkeit (AF), welche als Indikator für die multidimensionale Erholung der schwerverletzten Patienten (Funktionalität, psychische und physische Belastbarkeit) eingesetzt werden sollen. Die Sichtweise und Bedürfnisse der Betroffenen werden über patient-reported experience measure (PREM) einbezogen, welche im Projekt unter Einbeziehung der Expertise von Experten und ehemaligen Traumapatienten ebenso wie Kollektiv- und Projekt-spezifische patient-reported outcome measures (PROM) entwickelt werden. Mithilfe der Patienten- und Experteninterviews sollen zielgruppenorientierte Erhebungsprozesse erarbeitet werden, die, in den Studienkliniken eingesetzt, eine hohe Akzeptanz und Responserate im FollowUp von Schwerverletzten sichern sollen.

In dieser Mixed-Method Studie wird keine Intervention durchgeführt. Die einzelnen Datenquellen (GKV-Daten, Registerdaten, qualitative und quantitative Daten aus den Befragungen) werden nicht mit einander verknüpft. Ferner werden nur die Ergebnisse und

[1] Aus Gründen der besseren Lesbarkeit wird auf die gleichzeitige Verwendung der Sprachformen männlich, weiblich und divers (m/w/d) verzichtet. Sämtliche Personenbezeichnungen gelten gleichermaßen für alle Geschlechter.

Erkenntnisse, die sich aus den Analysen ergeben haben, zusammengeführt um Empfehlungen für die Versorgung zu identifizieren.

2.2 Art der Studie

- Medizinische, nicht interventionelle Forschung in Verbindung mit ärztlicher Versorgung
- Retrospektive anonymisierte Datenanalyse

2.3 Übersicht Studiendesign und Methodik

2.3.1 Qualitativer prospektiver Studienarm

Intersektorale und interprofessionelle Betrachtung des Behandlungsverlaufs durch semistrukturierte Interviews mit in der Versorgung involvierten Leistungserbringer und schwerst- und schwerverletzten Patienten.

2.3.2 Quantitative prospektive Kohortenstudie

Populationsbezogene, prospektive, multizentrische Kohortenstudie an schwerverletzten Patienten mit Baseline-Erhebung bei Entlassung und Befragung 6, 12 und 18 Monate nach schwerem Trauma.

2.3.3 Retrospektiver Studienarm

Aggregierte Ergebnisse aus Routinedaten des Wissenschaftlichen Instituts der AOK (WIdO) zur Charakterisierung von zeitlich-inhaltlichen poststationären Behandlungspfaden von Schwer- und Schwerstverletzten bis zur Wiedererlangung der Arbeitsfähigkeit über drei Jahre. Zusätzlich werden ergänzende anonymisierte Daten aus dem „TraumaRegister der Deutschen Gesellschaft für Unfallchirurgie (DGU)“ (beispielweise zu Verletzungen, Repräsentativität) hinzugezogen.

Hinweis

Fokus dieses Ethikantrags sind die Studienanteile *2.3.2 Quantitative prospektive Kohortenstudie* sowie *2.3.3 Retrospektiver Studienarm*. Für den Studienanteil *2.3.1 Qualitativer prospektiver Studienarm* wurde schon Anfang Mai ein Ethikantrag erreicht (Ethikantrag 1.0 qualitativer Studienarm), da die Ergebnisse dieser qualitativen

Interviews in die Erstellung des Fragebogens, der in *2.3.2 Quantitative prospektive Kohortenstudie* Verwendung findet, einfließen.

3. Verantwortlichkeiten

3.1 Verantwortliche Studienleiter und beteiligte Wissenschaftler

Die nachfolgende Tabelle (Tab. 1) listet alle an der Studie beteiligten Konsortialpartner auf und die involvierten Ärzte und wissenschaftlichen Mitarbeiter:

Tab. 1: Studienleiter und Wissenschaftler

**Name**	**Institution**	**Verantwortlichkeit/Rolle**
Dr.-Ing. Christine Höfer<br><br>Elisabeth Schwojer	AUC– Akademie der Unfall-chirurgie GmbH (AUC), München	Konsortialführung, Koordination u. Schulung Studienkliniken, Aufbau Studienregister
Prof. Dr. Rolf Lefering	Institut für Forschung in der Operativen Medizin (IFOM), Universität Witten/ Herdecke, Köln	Methodische Projektleitung, Biometrie
Prof. Dr .med. Joachim Windolf<br><br>Dr. med. Dan Bieler<br><br>Dr. med. Carina Jaekel<br><br>Anne Neubert	Klinik für Orthopädie und Unfallchirurgie, Universitätsklinikum Düsseldorf	Vorbereitung, Durchführung und Exploration Experten- und Patienteninterviews, Ableitung Maß-nahmen und Empfehlungen zur Verbesserung der Versorgung; Klinische Expertise
Prof. Dr. med. Felix Walcher<br><br>Gina Grimaldi<br><br>Susanne Drynda	Universitätsklinik für Unfallchirurgie, Otto-von-Guericke-Universität Magdeburg	Definition Studien-Assessments; Ableitung Maßnahmen und Empfehlungen zur Verbesserung der Versorgung, Klinische Expertise<br>Methodische Begleitung, Epidemiologie
Prof. Dr. med. M. Dudda<br><br>Dr. med. Oliver Kamp	Klinik für Unfall-, Hand- und Wiederherstellungschirurgie, Universitäts-Klinikum Essen	Erhebungsprozesse Follow-Up, Ableitung Maßnahmen und Empfehlungen zur Verbesserung der Versorgung, Klinische Expertise
Christian Günster	Wissenschaftliches Institut der AOK, Forschungsbereich Qualitäts- und Versorgungsforschung (WIdO)	Sekundärdatenanalyse, gesetzliche Krankenversicherung (AOK)

*unterstrichen, die jeweilige Studienleitung der jeweiligen Konsortialpartner

3.2 Finanzierung

Innovationsausschuss beim Gemeinsamen Bundesausschuss

Förderkennzeichen: 01VSF21033

Förderzeitraum: 01.04.2022 – 30.09.2025

3.3 Registrierung im Studienregister

Deutsches Register Klinischer Studien (DRKS): DRK S00028841

4. Wissenschaftlicher Hintergrund

In Deutschland erleiden jährlich circa 30.000 Menschen ein schweres Trauma (Jahresberichte des „TraumaRegister DGU"). In den letzten Jahrzehnten konnte durch zahlreiche Fortschritte in der präklinischen und klinischen Behandlung die Sterblichkeit von schwer- und schwerstverletzten Patienten deutlich gesenkt werden (Ruchholtz et al., 2008). Mit steigender Überlebensrate gewinnen nun das funktionelle Outcome und die gesundheitsbezogene Lebensqualität dieser Patienten an Bedeutung. Betroffene Patienten leiden unter anderem an chronischen Schmerzen, psychosomatischen Erkrankungen und Arbeitslosigkeit (Simmel et al., 2010; Simmel et al., 2013) sowie an gesteigerter posttraumatischer Morbidität (Bouillon et al.,1998; Holtslag et al., 2007; von Rüden et al., 2013).

Die Rückkehr der Patienten in die Arbeitswelt ist wichtig, um ihre Lebensqualität und soziale Teilhabe zu fördern und um die sozioökonomische Belastung der Gesellschaft durch Arbeitsunfähigkeit zu verringern. Nach aktuellen Zahlen des „TraumaRegister DGU" sind in Deutschland 60% der Schwer- und Schwerstverletzten im arbeitsfähigen Alter (18-65 Jahre), drei Viertel sind männlich. Bei einem gesetzlichen Renteneintritt von aktuell 67 Jahren kann sich bei Arbeitsunfähigkeit eine finanzielle Versorgungslücke von mehreren Jahrzehnten ergeben. Die hohe Rate an Arbeitslosigkeit nach Trauma ist bekannt. Laut der Hannover-Polytrauma-Langzeitstudie (HLPS) (Sittaro et al., 2007) sind durchschnittlich circa 22% der Patienten, welche zuvor arbeitsfähig waren, arbeitslos. Weitere 16% haben aufgrund ihres Unfalles zum Erhalt der Arbeitsfähigkeit eine Umschulung absolviert und bei 24% konnte eine unfallbedingte Frühberentung festgestellt werden. Einer niederländischen Studie zufolge konnten z. B. nur 58,5%, der nachuntersuchten schwer- und schwerstverletzten Patienten im arbeitsfähigen Alter, 15 Monate nach Trauma einer Vollzeitbeschäftigung und weitere 21,5% nur noch einer Teilzeitbeschäftigung nachgehen (Holtslag et al., 2007). Gemäß Daten aus einer Pilotstudie zur Outcomeerfassung sind in Deutschland nur etwa 30 % aller schwer- und schwerstverletzten Patienten zwei Jahre nach einem Unfall wieder arbeitsfähig (Kamp et al., 2019).

Folgende Prädiktoren der klinischen Behandlung für eine Erwerbsunfähigkeit konnten bereits identifiziert werden: Alter des Patienten, Schwere der Verletzung, Schädel-Hirn-Trauma, Dauer der intensivmedizinischen Behandlung, psychische Folgeerkrankungen, die subjektive Wahrnehmung der Verletzung und das Bildungsniveau des Patienten (Frink et al., 2014; Kaske et al., 2014).

Nicht untersucht wurde bisher die Frage, warum die Patienten nicht wieder arbeitsfähig bzw. arbeitsfähig aber unbeschäftigt sind. Ursachen könnten zum Beispiel organisatorischer Natur sein, z. B. Verzögerungen bei der Rehabilitation oder der Finanzierung einer Umschulung. Im Anschluss an die medizinische Akutversorgung in einem Traumazentrum schließt sich häufig eine Rehabilitationsmaßnahme an.

Über den Erfolg dieser Therapie entscheidet nicht die Dauer, sondern die Qualität der Rehabilitation (Simmel et al., 2009). Durch organisatorische Einschränkungen kommt es häufig zu einer Verzögerung der Maßnahme, der sog. Reha-Lücke. Ein validiertes Instrument zur Erfassung der Qualität der Rehabilitation existiert bisher nicht. Daher ist nicht evaluierbar, welche Maßnahmen und Hilfsmittel nach einem schweren Trauma im Durchschnitt benötigt werden. Auch hinsichtlich der Dauer der Rehabilitationsmaßnahme gibt es bislang wenig Evidenz. Der Zeitpunkt, zu dem die Rehabilitationsmaßnahme begonnen wird, scheint jedoch einen Einfluss zu haben.

Ein weiteres Problem besteht darin, dass nach der Entlassung von Schwer und Schwerstverletzten aus der akuten, stationären Behandlung ein patientenbezogenes Feedback zum langfristigen Outcome fehlt. Ohne derartiges Feedback endet die Qualitätssicherung der Akutklinik mit der Entlassung der Patienten und die Möglichkeiten der Verbesserung der Patientenversorgung beschränken sich auf die Bewertung des erreichten Entlasszustandes. Insbesondere bei Schwer- und Schwerstverletzten ist jedoch von einer langwierigen Erholungsphase auszugehen, bei der die akutstationäre Behandlung am Anfang einer langen Behandlungskette steht. Bereits durchgeführte Outcome-Studien beleuchten punktuell einzelne Fragestellungen (Janssen et al., 2008; Tecic et al., 2013). Die Darstellung der Versorgungsrealität während der gesamten Rekonvaleszenz und über die Sektorengrenzen hinweg ist jedoch, wie auch die Einbeziehung der Patientenerfahrungen und -bedürfnisse auf diesem Pfad, bisher ausstehend.

5. Projektziele

Das kurzfristige Projektziel ist die Identifizierung relevanter und beeinflussbaren Determinanten für gesundheitsbezogene Lebensqualität und Arbeitsfähigkeit in der Schwer- und Schwerstverletztenversorgung unter Einbezug von PREs und PROs. Langfristiges Ziel ist die Optimierung der Versorgungsqualität durch praxistaugliche Empfehlungen zu beeinflussbaren Faktoren.

5.1 Forschungsfragen des Gesamtprojekts

Folgende Forschungsfragen wurden für das Gesamtprojekt formuliert:

i. Wie stellt sich bei schwer- und schwerstverletzten Patienten die Realität in Bezug auf die Rückkehr in die Arbeitswelt zum aktuellen Zeitpunkt dar?
ii. Wer wird wann wieder arbeitsfähig?
iii. Welche Einflussfaktoren (z. B. Patient, Beruf, Unfallhergang, Verletzungsmuster und Therapie in verschiedenen Sektoren) gibt es in Bezug auf die Wiedererlangung der Arbeitsfähigkeit, und lassen sich diese beeinflussen?
iv. Wie entwickelt sich die gesundheitsbezogene Lebensqualität (hrQoL) nach einer schweren Verletzung im Zeitverlauf bis zur Wiederherstellung der Arbeitsfähigkeit?
v. Wodurch kennzeichnen sich günstige und ungünstige Patientenpfade und -verläufe?

5.2 Arbeitshypothesen des Projekts

Die nachfolgende Tabelle (Tab. 2) stellt die formulierten Hypothesen des gesamten LeAf-Projekts dar:

Tab. 2: Arbeitshypothesen des Gesamtprojekts

Hypothese 1	Es gibt beeinflussbare Risikofaktoren für die Wiedererlangung der Arbeitsfähigkeit im sektorenübergreifenden Behandlungsverlauf.
Hypothese 2	Diese Faktoren haben auch einen relevanten Anteil am post-traumatischen Zustand des Patienten im Hinblick auf die hrQoL.
Hypothese 3	PREMs helfen bei der Identifizierung von solchen Faktoren und bei der Erstellung von Empfehlungen und Maßnahmen zur Verbesserung der Patientenversorgung.

hrQoL = gesundheitsbezogene Lebensqualität; PREMs = patient-reported experience measures

6. Studienpopulation

Hinweis

Hier werden die Studienpopulationen der *2.3.2 Quantitative prospektive Kohortenstudie* sowie des Punktes *2.3.3 Retrospektiver Studienarm* beschrieben. Die Beschreibung der Studienpopulation für Punkt *2.3.1 Qualitativer prospektiver Studienarm* findet sich in Ethikantrag 1.0 - qualitativer Studienarm.

6.1 Einschluss- und Ausschlusskriterien

Die nachfolgende Tabelle (Tab. 3) stellt die formulierten Ein- und Ausschlusskriterien für die Studienpopulation dar.

Tab. 3: Ein- und Ausschlusskriterien

Studienteil	Einschlusskriterien	Ausschlusskriterien
Prospektive Kohortenstudie	• Schwerverletzte Patienten im arbeitsfähigen Alter (18-55 Jahre) mit einer Verletzungsschwere von einem MAIS* ≥3;	• Patienten, die nach Ende der Akutphase (Einschlusszeitpunkt) nicht kommunikationsfähig sind (Glasgow Outcome Score < 3)<br>• Keine hinreichenden Deutschkenntnisse (mindestens Sprachniveau B1 nach dem europäischen Referenzrahmen)<br>• Patienten nach Suizidversuch<br>• Patienten ohne festen Wohnsitz oder Wohnsitz im Ausland
Daten des TraumaRegister DGU	• Aufnahme eines Patienten über den Schockraum mit anschließender Intensivtherapie<br>• Alle im TR dokumentierten Patienten mit einem MAIS* ≥ 3,<br>• Akutaufenthalt überlebt<br>• Alter 18-55 Jahre<br>• Unfallzeitpunkt 2015-2017	• Patienten mit weniger schwerwiegenden Verletzungen<br>• Patienten nach Suizidversuch<br>• Patienten, die nach Ende der Akutphase (Einschlusszeitpunkt) nicht kommunikationsfähig sind (Glasgow Outcome Score < 3)
WIdO Sekundärdaten	• Versicherte im arbeitsfähigen Alter (18-55 Jahre)<br>• Schwere akutstationäre behandelte Verletzung in den Jahren 2015 bis 2017 überlebt haben und im Beobachtungszeitraum durchgängig versichert waren. Die hier angelegte Definition einer schweren Verletzung muss einem MAIS* von mindestens 3 (MAIS ≥ 3), bzw. einem ISS* ≥ 9 entsprechen..	•

	•	

** MAIS = maximalen Abbreviated Injury Scale; ISS = Injury Severity Score*

6.1.1 Weiterführende Hinweis in Bezug auf Ein- und Ausschlusskriterien

<u>TraumaRegister DGU:</u>
Es werden oftmals weniger schwer- und schwerstverletzte Patienten im TraumaRegister DGU dokumentiert. Das erhöht nicht nur den Dokumentationsaufwand, es erschwert auch die Vergleichbarkeit der Ergebnisse mit anderen Krankenhäusern oder im Zeitverlauf. Daher werden seit 2015 die Auswertungen nur noch für das Basiskollektiv durchgeführt und nicht mehr für alle dokumentierten Patienten.

<u>WIdO:</u>
Da über die Kodierrichtlinien des ICD-10GM *(= Internationale statistische Klassifikation der Krankheiten und verwandter Gesundheitsprobleme, German Modification)* keine Kodierung der MAIS oder des ISS möglich ist, werden zunächst die bereits bestehenden Identifikationskriterien basierend auf medizinischer Expertise überarbeitet, welche über Kombinationen aus spezifischen ICD-10GM Kodierungen und OPS-Kodierungen *(= Operationen- und Prozedurenschlüssel, Version 2019)* sowie DRGs in Sekundärdaten operationalisierbar sind. Die erste stationär behandelte Verletzung, die die Einschlusskriterien erfüllt, geht als Indexaufenthalt des Versicherten ein. Mit Entlassung aus dem Indexaufenthalt startet der dreijährige Nachbeobachtungszeitraum.

6.2 Anzahl der Studienteilnehmer

Die nachfolgende Tabelle 4 zeigt die Fallzahlplanung:

Tab. 4: Fallzahlplanung

**Studienteil**	**Fallzahl (n=)**
Prospektive Kohortenstudie	1000 schwer- und schwerstverletzte Patienten
WIdO Daten	33.000 schwer- und schwerstverletzte Versicherte
TraumaRegister DGU	29.345 (Basiskollektiv im Jahr 2019)

6.3 Fallzahlberechnung

6.3.1 Fallzahlberechnung prospektive Kohortenstudie

Da schwere Verletzungen in unterschiedlichen Körperregionen auftreten können, ist das vorliegende Kollektiv als heterogen anzusehen. Daher muss das Kollektiv groß genug sein, um neben patienten- und verletzungsspezifischen Subgruppenanalysen (zum Beispiel Schädel-Hirn-Trauma) auch sozioökonomische und psychosoziale Faktoren zu untersuchen. Bei einer Gesamtzahl von n=1000 auswertbaren Fällen (mit vollständigem FollowUp) können Prävalenzen von 10% mit einer Genauigkeit von ± 2% (95%Konfidenzintervall) angegeben werden, Prävalenzen von 20% mit ± 2,5%, und Prävalenzen von 50% mit ± 3%. In Subgruppen von 300 Fällen besteht immer noch eine Genauigkeit von ± 3,5/4,5/5,5% für die oben genannten Prävalenzen.

Für die multivariaten Analysen von Risikofaktoren sollten pro Faktor eine Zahl von 5-10 Patienten mit einem „Event“ (hier die Arbeitsfähigkeit) zur Verfügung stehen. Sollten ca. 30% der Patienten keine Tätigkeit (wieder) aufgenommen haben, könnten so im gesamten Kollektiv ca. 30 Prädiktoren untersucht werden, was hinreichend erscheint.

Es wird von einer Zustimmungsquote zur Teilnahme an der Studie von etwa 50% ausgegangen (Kaske, 2019), von denen erwartungsgemäß 25-30% das Follow-up nicht vollständig beenden. Nach Erfahrungswerten aus dem TraumaRegister DGU werden pro Jahr im Schnitt 120 Schwer- und Schwerstverletzte (MAIS ≥3) in einem Level-1-Traumazentrum in Deutschland behandelt. Nach Abzug der Verstorbenen (ca.10%) verbleiben rund 100 Patienten pro Jahr. Bei einer Teilnahmequote von 50% (n=50) und angenommenen maximalen Lost-to-Follow-up von 30% (n=35) sind etwa 30 teilnehmende Traumazentren notwendig, um 1000 auswertbare Patientenfälle in einer einjährigen Einschlussphase zu erhalten. Von den Studienkliniken wird zur Abschätzung eines Selektionsbias ein Screening Log der potentiellen Studienpatienten geführt. Zusätzlich erfolgt ein Abgleich der Patientencharakteristik mit dem Gesamtkollektiv des TraumaRegisters DGU unter Anwendung der gleichen Einschlusskriterien (Repräsentativität). Als Studienkliniken sollen als TraumaZentrum DGU zertifizierte Häuser eingebunden werden, die jährlich mindestens 40 Schwer- und Schwerstverletzte gemäß Einschlusskriterium TraumaRegister DGU behandeln. Derzeit haben bereits 45 TraumaZentren DGU mit jährlich 40-250 Patienten gemäß Einschlusskriterium dieser Studie ihr Interesse bekundet, als Studienklinik mitzuwirken, siehe Kooperationspartner. Es kann also davon ausgegangen werden, dass die Fallzahlen erreicht werden, ggf. sogar in kürzerer Einschlusszeit.

6.3.2 Fallzahlberechnung WIdO / TraumaRegister DGU

Im Jahr 2019 wurden in den bundesweiten WIdO-Daten rund 11.000 Versicherte im Alter von 18-55 Jahren mit einer akut stationär versorgten schweren Verletzung anhand der bereits bestehenden Identifikationskriterien identifiziert. Über den Einschusszeitraum von drei Jahren werden rund 33.000 schwer- und schwerstverletzte Versicherte in die Studie eingeschlossen werden können. Mit dieser angestrebten Fallzahl sind präzise Schätzungen von Prävalenzen auch innerhalb kleiner Subgruppen möglich. So ließe sich in einer Subgruppe von 5% der Gesamtzahl der Patienten eine Prävalenz von 50% mit einer Präzision von ±1,20% bis 1,38% (zweiseitiger exakter binomialer Konfidenzintervall 95%-Konfidenzintervall) schätzen. Eine Prävalenz von 50% wurde gewählt, da diese die größte Variation zeigt. Höhere oder niedrigere Prävalenzen sowie größere Subgruppen würden zu einer präziseren Schätzung führen.

6.4 Rekrutierungsmaßnahmen prospektive Kohortenstudie

Die Rekrutierung der Schwer- und Schwerstverletzen erfolgt über Kliniken des TraumaNetzwerk DGU während ihres Aufenthalts in der Studienklinik. Die Studienkliniken erhalten umfassende Schulungen zum Studienablauf und den FollowUp-Erhebungen (Prozesse, Inhalte). Die Studienkliniken sind zertifizierte TraumaZentren DGU und besitzen große Erfahrung in der Schwerverletztenversorgung und behandeln 50-250 Patienten/Jahr gemäß Einschlusskriterien der Studie (siehe oben).

7. Methodik und Durchführung

Zur Bearbeitung der Forschungsfragen verfolgt das Projekt einen quantitativen Ansatz bestehend aus zwei Kohorten von Schwer- und Schwerstverletzen, welcher um qualitative Methoden ergänzt wird. Kohorte 1 ist eine multizentrische, nicht-interventionelle, prospektive Studie mit dem Fokus auf klinischen Daten, PREs und PROs. In der Kohorte 2 liegt der Fokus auf der retrospektiven Betrachtung. Dafür werden GKV Routinedaten des Wissenschaftlichen Instituts der AOK (WIdO) zu Patientenpfaden bis hin zum Wiedereintritt ins Arbeitsleben vom WIdO selbst analysiert.

Qualitative Methoden sind im Ethikantrag 1 – qualitativer Studienarm beschrieben, diese dienen als Basis für Teile des Fragebogens der in der multizentrische, nicht-interventionelle, prospektive Kohortenstudie zum Einsatz kommt. Dieser Fragebogen wird in der Baseline Befragung um den TraumaRegister DGU Standardbogen (im Anhang) ergänzt sowie im FollowUp durch vorhandene Instrumente zur Befragung von Schwer- und Schwerstverletzten, die mittels Literaturrecherche identifiziert wurden, ergänzt.

7.1 Datenquellen

In der untenstehenden Abbildung 1 ist dargestellt, welche Datenquellen in LeAf Trauma zur Anwendung kommen. Die dort aufgeführten Patienten- und Experteninterviews sind ausführlich in Ethikantrag 1 – qualitativer Studienarm beschrieben. Aus der Abbildung wird die Wichtigkeit der Interviews für die anderen Studienarme deutlich: Insbesondere für die prospektive Kohortenstudie, aber auch für die Analyse der Sekundärdaten vom WIdO spielen die qualitativen Daten eine bedeutende Rolle. Unterhalb werden die drei übrigen Datenquellen, deren Methodik und deren Zusammenhänge ausführlich beschrieben.

Abb. 1: Datenquellen

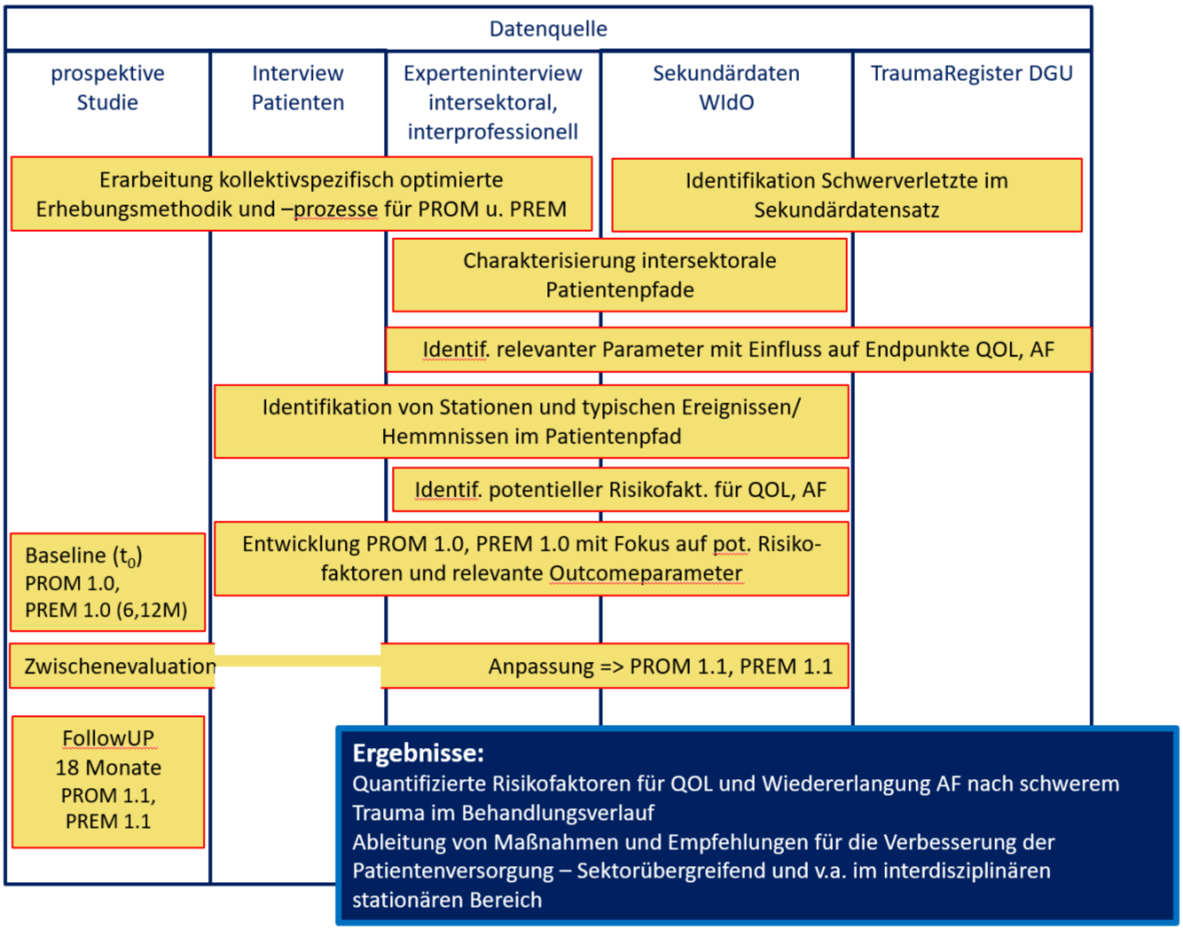

7.1.1 Im Vorfeld zur prospektiven Kohortenstudie

Die Anteile des Fragebogens, die aus den qualitativen Gruppeninterviews (beschrieben in Ethikantrag 1 – qualitativer Studienarm) hervorgegangen sind, sollen in einem Pretest auf Verständlichkeit und Usability evaluiert werden und ggf. eine Anpassung vor dem Einsatz in der Patientenversorgung erfahren (Version 1.0). Der Pretest wird mit austherapierten

Patienten aus dem Kollektiv, die an den Interviews teilgenommen haben, durchgeführt. Die FollowUp-Erhebungsprozesse werden auf Basis der Ergebnisse von Patienteninterviews, Literaturrecherche und Gesprächen mit Akteuren bereits durchgeführter FollowUp-Studien an Schwer- und Schwerstverletzten und unter Berücksichtigung von datenschutzrechtlichen und technischen Rahmenbedingungen entwickelt (siehe Ethikantrag 1 – qualitativer Studienarm).

7.1.2 Prospektive Kohortenstudie

Alle teilnehmenden Studienkliniken werden mindestens 2 Schulungen erhalten, in dem der Ablauf der Studie und des Follow Ups ausführlich erklärt werden. Die Rekrutierung, Administration, Nachverfolgung und Follow Up über den gesamten Studienverlauf erfolgt durch die jeweilige Studienklinik, die pro Patient eine nach Anzahl der erfolgreich in der webbasierten Studiendatenbank dokumentierten FollowUp gestaffelte Aufwandsentschädigung in Höhe von bis zu 660,00 € erhält. Die Falldokumentation aller Erhebungen erfolgt ausschließlich pseudonymisiert in der webbasierten Studiendatenbank. Die Zuordnungsliste wird von der jeweiligen Studienklinik für die eingeschlossenen Patienten verwaltet.

Nach Aufklärung und Einwilligung zur Studienteilnahme werden vor Entlassung die Baseline-Daten erhoben (klinische Daten, Beruf, Situation vor dem Unfall – genaue Inhalte basieren auf den Ergebnissen der Patienten- und Experteninterviews wie beschrieben in Ethikantrag 1 qualitativer Studienarm; weitere Informationen siehe auch Abschnitt Datenerhebung).

<u>FollowUp - Assessments</u>
FollowUp - Assessments erfolgen 6, 12 und 18 Monate nach Trauma unter anderem mit im Projekt zu erarbeitenden PREM und PROM (vgl. Abbildung 2). Die optimalen FollowUp - Erhebungsprozesse werden auf Basis der Ergebnisse Patienteninterviews, Literaturrecherche und Gesprächen mit Akteuren bereits durchgeführter FollowUp - Studien an Schwer- und Schwerstverletzten und unter Berücksichtigung von datenschutzrechtlichen und technischen Rahmenbedingungen entwickelt. Für das FollowUp werden die Patienten entweder postalisch oder telefonisch von den jeweiligen Studienkliniken befragt. Sie werden nicht von den Studienkliniken einbestellt. In einem initialen Arbeitspaket des Gesamtprojektes wird eine Patientengruppen-spezifische Erhebungsmethodik für FollowUp erarbeitet. Falls sich die postalische Abfrage als die beste Art der Befragung für diese Patientengruppe zeigt, werden die Rücksendungen an die jeweils behandelnde Studienklinik erfolgen. Weitere Partner erhalten keine Kenntnis von der Identität der Patienten.

Abb.2: Flussdiagramm zur prospektiven Kohortenstudie LeAf Trauma

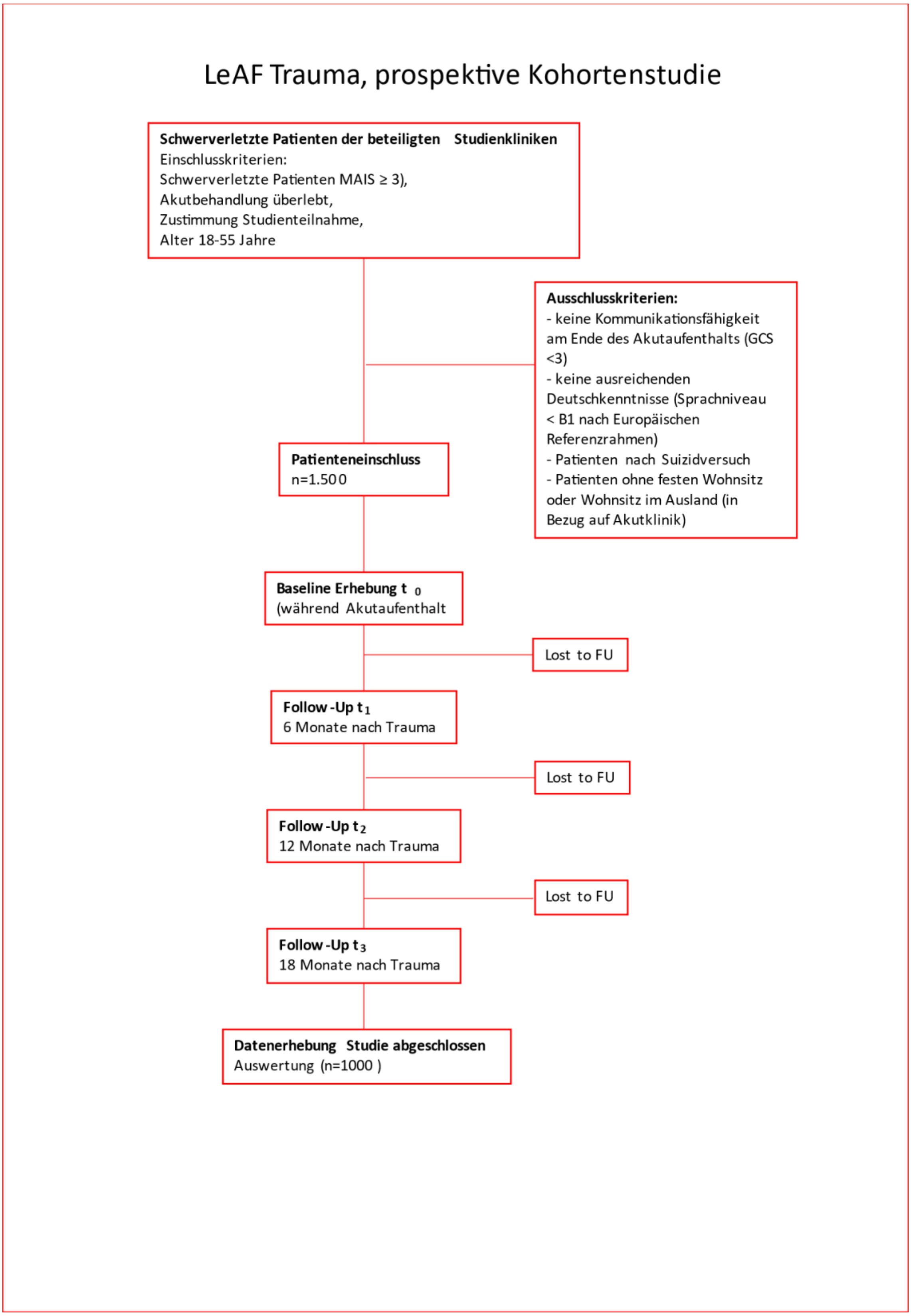

**FU = Follow Up, GCS = Glasgow Coma Scale, MAIS = maximal Abbreviated Injury Scale*

Zwischenevaluation

Es erfolgt eine Zwischenevaluation der Studienergebnisse (nach 6 Monats- und 12 Monats-FollowUp), in der die Ergebnisse (inhaltlich) aus den FollowUps analysiert werden und mit den Ergebnissen aus den Patienten- und Experteninterviews (siehe Ethikantrag 1 – qualitativer Studienanteil) abgeglichen werden sollen. Diese Ergebnisse werden dann ergänzt, um Erkenntnisse aus der Routinedatenanalyse (Oktober 2022 bis Juni 2023) und interdisziplinärer Bewertung derselben unter Hinzuziehen von intersektoralen Experten. Dies kann ggf. zu einer Erweiterung der im Fragebogen angewendeten PREMs und PROMs führen (Entwicklung Fragebogen Version 1.1), die dann im 18 Monats - FollowUp eingesetzt werden. Ziel der Zwischenevaluation ist es, herauszufinden, ob in den Gesprächen mit den Patienten weitere, für den Erholungsverlauf, die gesundheitsbezogene Lebensqualität, Wiedererlangung der Arbeitsfähigkeit relevante Aspekte aufgekommen sind, die im bisherigen Fragebogen nicht enthalten sind und als „Freitext“ ergänzend aufgenommen wurden. Daraus wird sich dann ggf. die Notwendigkeit ableiten, die PROMs und PREMs anzupassen. Eine angepasste Version der PROMs und PREMs werden voraussichtlich im 18-Monats FollowUp eingesetzt werden können.

Abb. 3: Ablauf prospektive Kohortenstudie

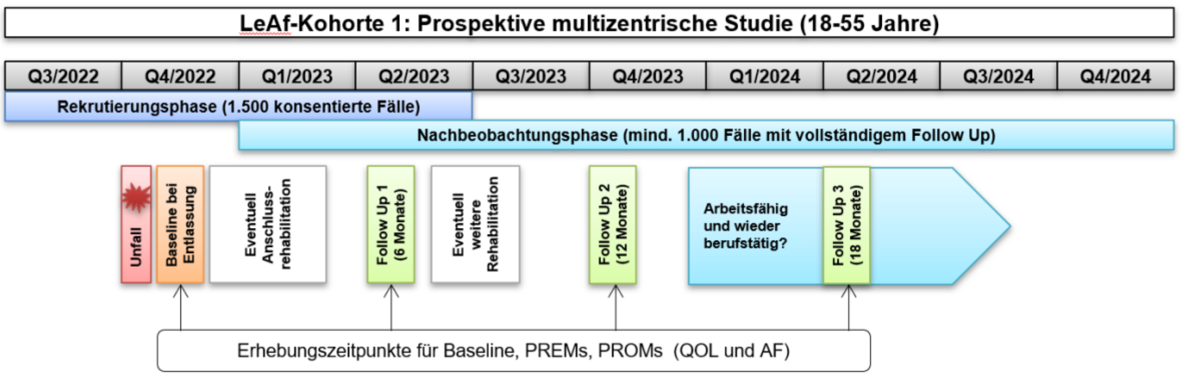

Nach Abschluss des Nachbeobachtungszeitraumes teilen die Mitwirkenden der Studienkliniken ihre Erfahrungen mit der Erhebung, des Patientenfeedbacks und ihrer persönlichen Einschätzung der Erhebungsprozesse über eine Onlinebefragung mit.

Die Auswertung der prospektiven Studie startet mit der Vorbereitung der Auswertealgorithmen im 4. Projektquartal (2023). Zwischenergebnisse fließen im Verlauf in die zeitliche und inhaltliche Charakterisierung von Patientenpfaden und der Versorgungsrealität von schwer- und schwerstverletzten GKV Patienten ein.

7.1.3 WIdO Daten

Die Sekundärdaten des Wido werden dazu genutzt, um eine Charakterisierung von zeitlich-inhaltlichen poststationären Behandlungspfaden von Schwer- und Schwerstverletzten bis zur Wiedererlangung der Arbeitsfähigkeit über drei Jahre darzustellen. Um schwer- und schwerstverletzte Patienten, deren Folgeerkrankungen und -behandlungen sowie deren Prädiktoren in Routinedaten identifizieren zu können, müssen basierend auf medizinischen Kenntnissen zunächst Definitionen erarbeitet werden, welche sich in GKV-Routinedaten operationalisieren lassen. Diese werden derzeit erarbeitet und dann dem WIdO übermittelt. Darüber hinaus werden für traumaspezifische Subgruppen ungünstige Krankheitsverläufe charakterisiert. Hierzu werden in medizinischen Expertengruppen mögliche Outcomes in definierten poststationären Zeiträumen als ungünstige Verläufe gekennzeichnet, bspw. Wiederaufnahmen, Infektionen, Pseudarthrose, Reoperationen. Die Wiedererlangung der Arbeitsfähigkeit als primäres Outcome sowie ungünstige Verläufe werden für zuvor als relevant identifizierte Subgruppen hinsichtlich möglicher Prädiktoren analysiert.
Ausschließlich das WIdO führt die Analyse dieser Kohorte durch, die anderen Konsortialpartner erhalten ausschließlich anonymisierte aggregierte Daten der definierten Kohorte. Diese Daten werden nicht mit anderen Projektdaten gelinkt und können nicht auf einen spezifischen Patienten zurückzuverfolgen sein. Das Wido wird hierfür bundesweite Daten der AOK der Jahre 2015 bis 2020 nutzen.

7.1.4 TraumaRegister DGU

Die Daten des TraumaRegisters DGU aus den Jahren 2015 bis 2017 dienen zur tiefergehenden Darstellung der Kohorte beispielsweise in Bezug auf Verletzungen, Repräsentativität. Im TraumaRegister DGU werden viele klinische Daten eines Schwer-und Schwerstverletzten dokumentiert. Dem Antrag wird der Standardbogen zur Erfassung der Patienten im TraumaRegister DGU beigelegt, der einen Überblick über die zu erfassenden Daten im TraumaRegister DGU gibt. Die Daten des TraumaRegisters DGU können ausschließlich über einen Antrag analysiert werden. Dieses Vorgehen ist über das Antrags- und Reviewverfahren gemäß Publikationsrichtlinien (wie hier beschrieben: https://www.traumaregister-dgu.de/forschung) geregelt. Die Daten werden dann ausschließlich in anonymisierter aggregierter Form in das Projekt eingebracht.

7.1.5 Datenerhebung prospektive Kohortenstudie (Baseline und FollowUp)

Die Patienten werden in dem prospektiven Arm der Studie insgesamt vier Mal (Baseline, 6, 12 und 18 Monats FollowUp) befragt. Zum Zeitpunkt der Entlassung aus der Studienklinik erfolgt eine Baseline-Befragung, im FollowUp folgt die Erhebung (Patient reported Outcomes (PROs)) zur gesundheitsbezogenen Lebensqualität (hrQoL) und Arbeitsfähigkeit

(AF) sowie die Auswertung des von dem Patienten erlebten Behandlungspfades (Patient Reported Experiences (PREs)). Die Erhebung der Patientendaten erfolgt über einen Fragebogen. Der Fragebogen wird aus mehreren Modulen zusammengesetzt. Es werden möglichst validierte Fragebögen eingesetzt. Die unter „Erfassung der Einflüsse auf die Arbeitsfähigkeit nach Trauma (PROM)“ und „Patient-Reported-Experience Measures (PREMs)“ aufgelisteten Messinstrumente werden in Abhängigkeit von den Patienten- und Experteninterviews ausgewählt und mit eigenständig konstruierten Fragen ergänzt. Die Bearbeitung des Fragebogens sollte nicht länger als 30 min dauern. Vor Beginn der Patientenrekrutierung wird der Fragebogen einem Pretest unterzogen und ggf. überarbeitet.

- TraumaRegister DGU Standardbogen (nur bei Baseline-Erhebung, siehe beigefügte Dokumente)

- Erhebung der Lebensqualität nach Trauma (PROMs)
    - POLO Chart

      Pirente, N., Bouillon, B., Schäfer, B. *et al.* Systematische Entwicklung eines Messinstruments zur Erfassung der gesundheitsbezogenen Lebensqualität beim polytraumatisierten Patienten Die Polytrauma-Outcome- (POLO) Chart. *Unfallchirurg* **105,** 413–422 (2002). https://doi.org/10.1007/s00113-001-0348-5

- Erfassung der Einflüsse auf die Arbeitsfähigkeit nach Trauma
    - Gesundheitsbezogene Faktoren (physisch und psychisch)
    - Soziodemografische Faktoren
    - Personenbezogene Faktoren und Persönlichkeitsstruktur
    - Rehabilitationsbezogene Faktoren
    - Arbeitsplatzbezogene Faktoren
    - Verletzungsmerkmale und Informationen bezüglich des Krankenhausaufenthaltes

- Patient-Reported-Experience (PREMs)
    - Umgang und Behandlung in der Akutklinik
    - Beurteilung der medizinischen Schnittstellen im Patientenpfad
    - Beschreibung der organisatorischen Hürden mit Behörden und Institutionen

Voraussichtlich werden in den Patienten- und Experteninterviews weitere Merkmale der Patientenerfahrung im Behandlungspfad herausgestellt werden können, diese werden bei der Konstruktion des Fragebogens berücksichtigt.

7.2 Statistische Analyse

Beschreibung des Verlaufs: Alle Patienten; Beschreibung der Wiedererlangung der Arbeitsfähigkeit (AF) und gesundheitsbezogene Lebensqualität im Zeitverlauf (3 Follow Up Zeitpunkte; deskriptive Statistik).

Prädiktoren für AF: AF bis 18 Monate nach Trauma als ja/nein Kriterium; univariate und multivariate Analyse (logistische Regression) möglicher Prädiktoren; Einteilung der Prädiktoren in die Kategorien: Patient, Beruf, Präklinik, Klinik, postakute Therapie, Vorhandensein psychischer Probleme (aus QoL Assessment zum Follow Up Zeitpunkt).

Prädiktoren für Zeit bis AF: Nur Patienten mit Wiedererlangung der AF: Zeit bis AF als abhängige Variable, Kaplan-Meier Kurven zur Deskription, Cox-Regression zur multivariaten Analyse, Prädiktoren wie oben.

gesundheitsbezogene Lebensqualität und PREMs: Deskription zu den 3 Follow Up Zeitpunkten, Korrelation mit AF als Subgruppenvergleiche zu jedem Messzeitpunkt.

8. Freiwilligkeit und Rücktritt

Die Teilnahme der Patienten an der Studie erfolgt freiwillig. Die Zustimmung kann von den Teilnehmern jederzeit, ohne Angaben von Gründen und ohne persönliche Nachteile ganz oder in Teilen zurückgezogen werden. Die Studienteilnehmer werden vor Studienbeginn schriftlich und mündlich über das Vorgehen der Studie und über den Datenschutz aufgeklärt. Ihre Zustimmung wird durch Unterschrift auf der Einwilligungserklärung dokumentiert. Bei Rücktritt von der Studie wird bereits gewonnenes Datenmaterial vernichtet, es sei denn, der Studienteilnehmer ist mit der Auswertung des Materials einverstanden.

Bei der Aufklärung der Patienten und Einholung der Einwilligung werden die Patienten zusätzlich gebeten, der wissenschaftlichen Sekundärnutzung der im Rahmen des LeAf Trauma Projektes erhobenen Studiendaten zuzustimmen. Diese Zustimmung ist optional und hat keinerlei Auswirkung auf die Teilnahme an der propektiven multizentrischen Kohortenstudie.

Da alle beteiligten Studienkliniken als TraumaZentren DGU zertifiziert sind, sind diese vertraglich verpflichtet, Patienten, die die Einschlusskriterien des TraumaRegisters DGU treffen und ihre Einwilligung erteilt haben, ins TraumaRegister DGU einzuschließen.

Deshalb werden Patienten, die sowohl die Einschlusskriterien für die prospektive LeAf Trauma Kohortenstudie als auch für das TraumaRegister DGU treffen, zusätzlich um ihre optionale Einwilligung zur Teilnahme am TraumaRegister DGU gebeten. Im TraumaRegister werden ausschließlich Daten der Routinedokumentation im Klinikum erfasst. Es werden keine zusätzlichen Daten erhoben. Eine Teilnahme am TraumaRegister ist freiwillig und hat keine Auswirkung auf die Möglichkeit einer Teilnahme an der LeAf Trauma Studie.

9. Unerwünschte Wirkungen und Belastungen für Teilnehmer

Die Verarbeitung und Nutzung der Primärdaten aus der prospektiven Kohortenstudien soll unter Berücksichtigung der Integrität (z.B. Schutz vor vorsätzlicher oder fahrlässiger Verfälschung von Programmen oder Manipulation von Daten), der Vertraulichkeit (z.B. Schutz vor unbefugter Kenntnisnahme von Daten) und der Verfügbarkeit (z.B. Schutz vor Diebstahl oder Zerstörung) gewährleistet werden. Als weiterer zentraler Punkt wird die Notwendigkeit gesehen, Daten zu pseudonymisieren. Über aktuelle technische und organisatorische Schutzmaßnahmen, siehe Datenschutzkonzept zur prospektiven Kohortenstudie wird der Schutz der Patientendaten sichergestellt, Somit sind die Datenschutzrisiken als niedrig anzusehen.

Eine psychische Belastung durch aktives Erinnern an das Trauma/den Heilungsverlauf ist möglich, jedoch haben die teilnehmenden Studienkliniken sehr viel Erfahrung in der Behandlung von Schwer- und Schwerstverletzten (jährlich zwischen 40 – 250 Patienten). Des Weiteren sind sich die wissenschaftlichen Mitarbeiter des Projekts der Sensibilität des Gesprächsthemas bewusst und informieren und schulen die teilnehmen Studienkliniken in den geplanten Schulungen auch nochmals für dieses Thema.

10. Nutzen-Risiko Analyse

Der persönliche Nutzen durch die Teilnahme ist als sehr gering einzuschätzen, jedoch ist der Zeitaufwand für die Patienten ebenfalls gering.
Es gibt ein potenzielles Risiko für psychische Belastung der Patienten, wie unter „Komplikationen“ erläutert. Dem entgegen steht das hohe Verwertungspotential der gewonnenen Erkenntnisse in mehrfacher Hinsicht, wie beispielweise:

- Die Unverzichtbarkeit der Ergebnisse in der Entwicklung von Empfehlungen für die Verbesserung der Versorgung von Schwer- und Schwerstverletzten - Dies setzt unmittelbar an der Versorgungsrealität an und soll dazu genutzt werden, Hemmnisse für

den Wiedereintritt in das Arbeitsleben zu identifizieren und ggf. abzubauen und die Versorgung zu verbessern

- Die Bedeutung der Ergebnisse für alle an der Versorgung von schwer- und schwerstverletzten Beteiligten, da die Ergebnisse für Qualitätsprobleme in der Versorgung sensibilisieren, Versorgungspfade optimieren können und neue Kooperationen – interprofessionell und intersektoral – ebnen können
- Verbesserungsmaßnahmen in der Akutphase der Behandlung von Schwer- und Schwerstverletzten sollen direkt in flankierende Projekte und Initiativen der DGU inhaltlich einfließen – z.B. die Zertifizierung von TraumaZentren und TraumaNetzwerken DGU sowie die Kooperation zwischen TraumaNetzwerk und Rehabilitationskliniken
- Die generelle Relevanz der Ergebnisse für Aus-, Weiter- und Fortbildung von Professionellen in der Versorgung von Schwer- und Schwerstverletzten
- Verbesserung der Versorgung von zukünftigen Schwer- und Schwerstverletzten

11. Datenmanagement und Datenschutz

Im Abschnitt Datenmanagement und Datenschutz wird sich auf die prospektive Kohortenstudie bezogen. Die retrospektiven Daten liegen ausschließlich aggregiert und anonymisiert vor (GKV Routinedaten werden vom WIdO selbst analysiert. Zur Analyse der TraumaRegister Daten wird ein Antrag wie oben beschrieben gestellt (das Antrags- und Reviewverfahren gemäß Publikationsrichtlinien (https://www.traumaregister-dgu.de/forschung), die Daten werden ausschließlich in aggregierter anonymisierter Form innerhalb des Konsortiums verwertet und publiziert. Sie finden weitere Informationen zum Datenschutz im beigefügten Datenschutzkonzept.

Die Patientendaten werden in einem zentralen Studienregister gespeichert. Die Datenübermittlung in das Studienregister erfolgt webbasiert über eine gesicherte Verbindung (https) nach Login mit Benutzernamen und Passwort durch das eingebundene, medizinische Personal. Betreiber des Studienregisters ist die Projektleitung die AUC. Die Datenverarbeitung erfolgt nach einem ausführlichen Rechte- und Rollenkonzept. Details sind im Datenschutzkonzept einzusehen, dieses befindet sich in den Anhängen.

Ein differenziertes Einwilligungsmanagement mit festgelegten Dokumentationskonzepten gewährt Transparenz und Information über die Intervenierbarkeit. Die Patienten können die Wahrnehmung ihrer Betroffenenrechte jederzeit gegenüber der Studienleitung oder gegenüber jedem weiteren Konsortialpartner als verantwortliche Stelle oder gegenüber der behandelnden Studienklinik artikulieren.

Die Datenerhebung und -verarbeitung umfasst zur Wahrung der Zweckbindung ein abgestuftes Rollen- und Zugriffsrechtekonzept nach dem Erforderlichkeitsprinzip auf Basis eines Identitätsmanagements Anwendung. Die Autorisierung initialer Benutzer des Studienregisters von LeAf Trauma von Seiten der Studienkliniken erfolgt nach Vertragsabschluss über die AUC. Die Authentifizierung von Benutzern des Studienregisters erfolgt über Benutzername und Passwort. Das eingesetzte Personal ist eingegrenzt auf nachprüfbar zuständige, fachlich befähigte und formal zugelassene Personen. Diese sind zur Wahrung des Datengeheimnisses vertraglich bzw. aufgrund ihres Berufes zur Verschwiegenheit verpflichtet. Ein sicheres Authentifizierungsverfahren mittels Benutzername und Passwort ist implementiert.

Abb. 4: Datenflüsse und Verarbeitungsschritte in der prospektiven Kohortenstudie im Rahmen des LeAf Trauma Projekts

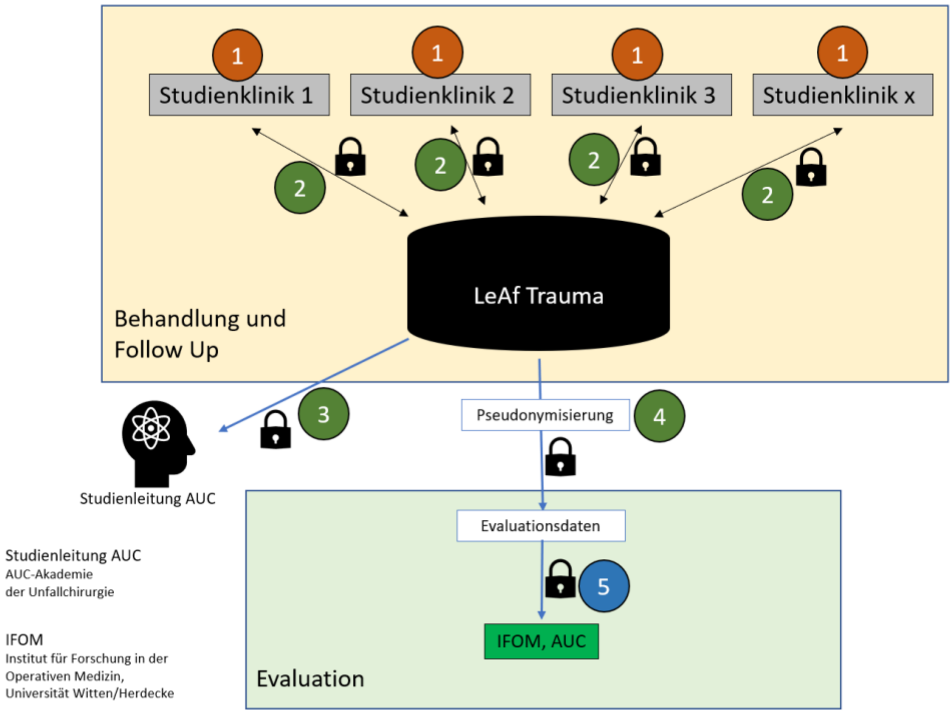

1 Einschluss Patienten, Erhebung Gesundheitsdaten
- Erhebung Stammdaten, Kontaktdaten
- Erfassung Anamnesedaten, Diagnostikdaten, Diagnosedaten, Behandlungsdaten und Outcomedaten
- Erhebung Lebensqualität und Status berufliche Betätigung

2 Pseudonymisierung und verschlüsselte Übermittlung der erhobenen Patientendaten in das Studienregister
- Pseudonymisierung
- Übermittlung
- Löschen von Datensätzen bei Widerruf der Einwilligung
- Datenkorrektur, sofern erforderlich

3 Kontinuierliche Überprüfung der Eingabequalität zur Sicherung der Datenintegrität und -vollständigkeit

4 erneute Pseudonymisierung (Patientenebene, Klinikebene)

5 Übermittlung und Offenlegung pseudonymisierte Patientendaten

→ Erhebung, Übermittlung und Zugriff
→ Übermittlung und Offenlegung

Behandlung und Follow Up
Evaluation

Personenbezogene Daten, Gesundheitsdaten

Pseudonymisierte Gesundheits-/Daten mit Klinikbezug

Pseudonymisierte Evaluationsdaten

Studienleitung (AUC)

Quellen

Bouillon B, Neugebauer E. Outcome after polytrauma. *Langenbeck's Arch Surg* (1998) 383:228-34

Frink M, Ruchholtz S, Debus F. Long-term results after major injury. OUP 2014; 11;512.515, DOI 10.3238/oup.20214.0512-0515

Holtslag HR, Post MW, van der Werken C, Lindemann E. Return to work after major trauma. *Clinical Rehabilitation* (2007) 21:373-83

Janssen C, Ommen O, Neugebauer E, Lefering R, Pfaff H. Predicting health-related quality of life of severely injured patients: sociodemographic, economic, and hospital stay-related determinants. *Europ. J. Trauma Emerg. Med.* 2008, 34: 277-286

Kamp O, Pfeifer R, Ritschel M, Flohe S, Bieler D. Polytrauma outcome: implementation of health-related quality of life assessment into the German Trauma Registry. *Europ. J. Trauma Emerg. Med.* (2019) online first

Kaske S, Lefering R, Trentzsch H, Driessen A, Bouillon B, Maegele M, Probst C. Quality of life two years after severe trauma: A single centre evaluation. *Injury* 2014 Suppl. S45: S100-S105

Ruchholtz S, Lefering R, Paffrath T, Oestern HJ, Neugebauer E, Nast-Kolb D, Pape HC, Bouillon B. Reduction in mortality of severely injured patients in Germany. Dtsch Arztebl Int. 2008 Mar; 105(13):225-31.

Simmel S, Frührehabilitation nach Polytrauma. Trauma Berufskrankh (2010) 12[Suppl 2]:194–198

Simmel S, Bühren V. Unfallfolgen nach schweren Verletzungen. Konsequenzen für die Trauma-Rehabilitation. *Chirurg* (2013) 84:764-70

Sittaro NA, Lohse R. Hannover-P olytrauma-Langzeitstudie (HPLS) Schriftenreihe zu aktuellen Themen der Schadenversicherung, e+s Rück, 2007

Tecic T, Lefering R, Althaus A, Rangger C, Neugebauer E. Pain and quality of life one year after admission to emergency department: Factors associated with pain. *Europ. J. Trauma Emerg. Surg.* 2013; 39: 353-61

von Rüden C, Woltmann A, Röse M, Wurm S, Rüger M, Hierholzer C, Bühren V. Outcome after severe multiple trauma: a retrospective analysis. *J. Trauma Manag. Outcome* (2013) 7:4
