# Supplementary material for: LeAf Trauma- an intersectoral prospective multicenter study assessing quality of life and return to work after majortrauma–study protocol
Source: PLoS One. 2024 Nov 13;19(11):e0312320. doi: 10.1371/journal.pone.0312320 (PMC11560036; doi:10.1371/journal.pone.0312320)
Supplement: S5 File — (PDF) [file pone.0312320.s005.pdf]

# S4 - Study Protocol_translation

**LeAf Trauma - Quality of Life and Work Ability after Severe Trauma - Quantitative Study Components**

**Version 1.0 – June 22, 2022**

## Table of Contents


1. Project Title 2
2. Project Summary 2
3. Responsibilities 4
4. Scientific Background 5
5. Project Objectives 7
6. Study Population 7
7. Methodology and Implementation 11
8. Voluntariness and Withdrawal 18
9. Adverse Effects and Burdens for Participants 19
10. Benefit-Risk Analysis 19
11. Data Management and Data Protection 20

    Sources 23


## 1. Project Title

**"LeAf Trauma - Quality of Life and Work Ability after Trauma" - Quantitative Study Components**

## 2. Project Summary

### 2.1 Scientific Description

The outcome of severely injured patients depends on multiple factors. The treatment pathway is characterized by transitions between sectors (e.g., emergency services > hospital > rehabilitation > outpatient follow-up) and interdisciplinary treatment that supports various aspects of recovery. The project "LeAf Trauma" aims to identify modifiable risk factors and quantify their influence to derive measures and recommendations for improving patient care and reducing barriers in the patient pathway. The patient-relevant endpoints are health-related quality of life (hrQoL) and the restoration of work ability (AF), which serve as indicators of the multidimensional recovery of severely injured patients (functionality, psychological and physical resilience). The perspectives and needs of the affected individuals will be incorporated through patient-reported experience measures (PREM), which will be developed in the project with the expertise of experts and former trauma patients, as well as collective and project-specific patient-reported outcome measures (PROM). Through patient and expert interviews, target-oriented data collection processes will be developed, which, when implemented in the study hospitals, should ensure high acceptance and response rates in the follow-up of severely injured patients.

This mixed-method study does not involve any intervention. The individual data sources (statutory health insurance data, registry data, qualitative and quantitative data from surveys) will not be linked. Furthermore, only the results and findings derived from the analyses will be compiled to identify recommendations for care.

### 2.2 Type of Study

- Medical, non-interventional research in conjunction with medical care
- Retrospective anonymized data analysis

### 2.3 Overview of Study Design and Methodology

**2.3.1 Qualitative Prospective Study Arm** Intersectoral and interprofessional examination of the treatment process through semi-structured interviews with healthcare providers involved in the care of severely injured patients.

**2.3.2 Quantitative Prospective Cohort Study** Population-based, prospective, multicenter cohort study involving severely injured patients, with baseline data collected at discharge and follow-up surveys at 6, 12, and 18 months post-trauma.

**2.3.3 Retrospective Study Arm** Aggregated results from routine data of the Scientific Institute of the AOK (WIdO) to characterize the temporal and content-related post-hospital treatment pathways of severely injured patients until the restoration of work ability over three years. Additionally, supplementary anonymized data from the "Trauma Register of the German Society for Trauma Surgery (DGU)" (for example, regarding injuries, representativeness) will be included.

## 3. Responsibilities

### 3.1 Responsible Study Leaders and Involved Scientists

The following table (Tab. 1) lists all consortium partners involved in the study, as well as the physicians and scientific staff involved:

**Tab. 1: Study Leaders and Scientists**

| Name | Institution | Responsibility/Role |
|---|---|---|
| Dr.-Ing. Christine Höfer | AUC – Academy of Trauma Surgery GmbH (AUC), Munich | Consortium leadership, coordination and training of study hospitals, establishment of study registry |
| Prof. Dr. Rolf Lefering | Institute for Research in Operative Medicine (IFOM), University of Witten/Herdecke, Cologne | Methodological project leadership, biostatistics |
| Prof. Dr. med. Joachim Windolf | Clinic for Orthopedics and Trauma Surgery, University Hospital Düsseldorf | Preparation, implementation, and exploration of expert and patient interviews; deriving measures and recommendations for improving care; clinical expertise |
| Dr. med. Dan Bieler | | |

| Name | Institution | Responsibility/Role |
|---|---|---|
| Dr. med. Carina Jaekel | | |
| Anne Neubert | | |
| Prof. Dr. med. Felix Walcher | University Hospital for Trauma Surgery, Otto von Guericke University Magdeburg | Definition of study assessments; deriving measures and recommendations for improving care; clinical expertise; methodological support, epidemiology |
| Prof. Dr. med. M. Dudda | Clinic for Trauma, Hand, and Reconstructive Surgery, University Hospital Essen | Data collection processes for follow-up; deriving measures and recommendations for improving care; clinical expertise |
| Dr. med. Oliver Kamp | | |
| Christian Günster | Scientific Institute of the AOK, Research Area for Quality and Healthcare Research (WIdO) | Secondary data analysis, statutory health insurance (AOK) |

*Underlined names indicate the respective study leadership of the respective consortium partners.*

### 3.2 Funding

Innovation Committee at the Joint Federal Committee

### 3.3 Registration in the Study Register

German Clinical Trials Register (DRKS): DRKS00028841

## 4. Scientific Background

In Germany, approximately 30,000 people suffer a severe trauma each year (annual reports of the "Trauma Register DGU"). In recent decades, significant progress in preclinical and clinical treatment has markedly reduced the mortality rate of severely injured patients (Ruchholtz et al., 2008). With increasing survival rates, the functional outcomes and health-related quality of life of these patients are becoming more important. Affected patients suffer from chronic pain, psychosomatic disorders, and unemployment (Simmel et al., 2010; Simmel et al., 2013), as well as increased post-traumatic morbidity (Bouillon et al., 1998; Holtslag et al., 2007; von Rüden et al., 2013).

The return of patients to the workforce is important to promote their quality of life and social participation and to reduce the socioeconomic burden on society due to disability. According to current data from the "Trauma Register DGU," 60% of severely injured patients in Germany are of working age (18-65 years), and three-quarters are male. With a current legal retirement age of 67 years, disability can lead to a financial gap of several decades. The high unemployment rate after trauma is well-known. According to the Hannover Polytrauma Long-Term Study (HLPS) (Sittaro et al., 2007), an average of about 22% of patients who were previously able to work are unemployed. Another 16% have undergone retraining due to their

accident to maintain work ability, and 24% have been classified for early retirement due to the accident. According to a Dutch study, only 58.5% of the severely injured patients of working age who were followed up were able to engage in full-time employment 15 months after the trauma, and an additional 21.5% could only pursue part-time employment (Holtslag et al., 2007). According to data from a pilot study on outcome assessment, only about 30% of all severely injured patients in Germany are able to work again two years after an accident (Kamp et al., 2019).

Following predictors of clinical treatment for disability have already been identified: patient age, severity of injury, traumatic brain injury, duration of intensive medical treatment, psychological sequelae, subjective perception of the injury, and the patient’s education level (Frink et al., 2014; Kaske et al., 2014).

However, the question of why patients are not able to return to work or are able to work but unemployed has not yet been investigated. Causes could be of an organizational nature, such as delays in rehabilitation or funding for retraining. Following acute medical care in a trauma center, rehabilitation measures often follow.

The success of this therapy is determined not by its duration but by the quality of the rehabilitation (Simmel et al., 2009). Organizational constraints often lead to delays in the rehabilitation measures, creating what is known as the rehabilitation gap. Currently, there is no validated instrument for assessing the quality of rehabilitation. Therefore, it is not possible to evaluate which measures and aids are typically needed after a severe trauma. There is also little evidence regarding the duration of the rehabilitation measures. However, the timing of when the rehabilitation measure begins seems to have an impact.

Another issue is that there is a lack of patient-specific feedback on long-term outcomes after the discharge of severely injured patients from acute inpatient care. Without such feedback, quality assurance in the acute clinic ends with the patient’s discharge, and opportunities to improve patient care are limited to assessing the achieved discharge status. However, particularly for severely injured patients, a lengthy recovery phase is to be expected, in which acute inpatient treatment is just the beginning of a long treatment chain. Previous outcome studies have addressed specific questions (Janssen et al., 2008; Tecic et al., 2013). However, the depiction of the care reality throughout the entire recovery period and across sector boundaries, as well as the inclusion of patient experiences and needs along this path, is still pending.

## 5. Project Objectives

The short-term project goal is to identify relevant and modifiable determinants for health-related quality of life and work ability in the care of severely injured patients, incorporating patient-reported experiences (PREs) and patient-reported outcomes (PROs). The long-term goal is to optimize the quality of care through practical recommendations on modifiable factors.

### 5.1 Research Questions of the Overall Project

The following research questions have been formulated for the overall project:

i. What is the current reality for severely injured patients regarding their return to the workforce?

ii. Who will be able to return to work, and when?
iii. What influencing factors (e.g., patient, occupation, accident circumstances, injury patterns, and therapy across different sectors) exist concerning the restoration of work ability, and can these be influenced?
iv. How does health-related quality of life (hrQoL) develop over time following a severe injury until work ability is restored?
v. What characterizes favorable and unfavorable patient pathways and trajectories?

### 5.2 Working Hypotheses of the Project

The following table (Tab. 2) presents the formulated hypotheses of the entire LeAf project:

**Tab. 2: Working Hypotheses of the Overall Project**

| | |
|---|---|
| Hypothesis 1 | There are modifiable risk factors for the restoration of work ability in the cross-sector treatment process. |
| Hypothesis 2 | These factors also play a relevant role in the post-traumatic condition of the patient regarding hrQoL. |
| Hypothesis 3 | PREMs assist in identifying such factors and in developing recommendations and measures to improve patient care. |

*hrQoL = health-related quality of life; PREMs = patient-reported experience measures*

## 6. Study Population

## 6.1 Inclusion and Exclusion Criteria

The following table (Tab. 3) presents the formulated inclusion and exclusion criteria for the study population.

**Tab. 3: Inclusion and Exclusion Criteria**

| Study Part | Inclusion Criteria | Exclusion Criteria |
|---|---|---|
| **Prospective Cohort Study** | • Severely injured patients of working age (18-55 years) with an injury severity of MAIS* ≥3; | • Patients who are not communicative after the acute phase (inclusion point) (Glasgow Outcome Score < 3)<br>• Insufficient German language skills (at least B1 language level according to the European Reference Framework)<br>• Patients after a suicide attempt<br>• Patients without a permanent residence or residing abroad |
| **DGU Trauma Register** | • Admission of a patient via the emergency room with subsequent intensive therapy | • Patients with less severe injuries |

| Study Part | Inclusion Criteria | Exclusion Criteria |
| --- | --- | --- |
| | • All patients documented in the TR with a MAIS* $\geq 3$, | • Patients after a suicide attempt |
| | • Acute stay survived | • Patients who are not communicative after the acute phase (inclusion point) (Glasgow Outcome Score < 8) |
| | • Age 18-55 years | |
| | • Accident date 2015-2017 | |
| **WIdO Secondary Data** | • Insured individuals of working age (18-55 years) | |
| | • Severe acute hospital-treated injury survived between 2015 and 2017 and continuously insured during the observation period. The defined severe injury must correspond to a MAIS* of at least 3 (MAIS $\geq 3$) or an ISS* $\geq 9$. | |

*MAIS = Maximum Abbreviated Injury Scale; ISS = Injury Severity Score

### 6.1.1 Further Notes Regarding Inclusion and Exclusion Criteria for DGU Trauma Register:

Less severely and severely injured patients are often documented in the DGU Trauma Register. This not only increases the documentation effort but also complicates the comparability of results with other hospitals or over time. Therefore, since 2015, evaluations have only been conducted for the base collective and no longer for all documented patients.

**WIdO:**
Since the coding guidelines of ICD-10GM (= International Statistical Classification of Diseases and Related Health Problems, German Modification) do not allow for coding of MAIS or ISS, the existing identification criteria based on medical expertise will first be revised. These will be operationalizable in secondary data through combinations of specific ICD-10GM codes, OPS codes (= Operation and Procedure Key, version 2019), and DRGs. The first hospitalized injury that meets the inclusion criteria will be considered the index stay of the insured. The three-year follow-up period starts with the discharge from the index stay.

## 6.2 Number of Study Participants

The following table (Tab. 4) shows the planned sample size:

**Tab. 4: Sample Size Planning**

| Study Part | Sample Size (n=) |
| --- | --- |
| **Prospective Cohort Study** | 1000 severely and critically injured patients |
| **WIdO Data** | 33,000 severely and critically injured insured |
| **DGU Trauma Register** | 29,345 (Base collective in 2019) |

## 6.3 Sample Size Calculation

### 6.3.1 Sample Size Calculation for Prospective Cohort Study

As severe injuries can occur in different body regions, the present cohort is considered heterogeneous. Therefore, the cohort must be large enough to allow for analyses of patient- and injury-specific subgroups (for example, traumatic brain injury), as well as the examination of socioeconomic and psychosocial factors. With a total of n=1000 evaluable cases (with complete follow-up), prevalences of 10% can be reported with an accuracy of ± 2% (95% confidence interval), prevalences of 20% with ± 2.5%, and prevalences of 50% with ± 3%. In subgroups of 300 cases, there is still an accuracy of ± 3.5/4.5/5.5% for the aforementioned prevalences.

For the multivariate analyses of risk factors, a number of 5-10 patients per factor with an "event" (in this case, work ability) should be available. If approximately 30% of patients have not resumed work, about 30 predictors could be investigated across the entire cohort, which seems sufficient.

It is assumed that the consent rate for participation in the study will be about 50% (Kaske, 2019), of which it is expected that 25-30% will not complete the follow-up. According to experience from the DGU Trauma Register, an average of 120 severely and critically injured patients (MAIS ≥3) are treated annually in a Level-1 trauma center in Germany. After accounting for deceased patients (approximately 10%), around 100 patients remain per year. With a participation rate of 50% (n=50) and an assumed maximum lost-to-follow-up rate of 30% (n=35), approximately 30 participating trauma centers will be necessary to obtain 1000 evaluable patient cases within a one-year inclusion phase. To estimate selection bias, the study clinics will maintain a screening log of potential study patients. Additionally, a comparison of patient characteristics with the overall cohort of the DGU Trauma Register will be made using the same inclusion criteria (representativeness). The study clinics will include DGU-certified trauma centers that treat at least 40 severely and critically injured patients annually according to the inclusion criteria of the DGU Trauma Register. Currently, 45 trauma centers have already expressed interest in participating as study clinics, treating 40-250 patients annually according to the inclusion criteria of this study, see cooperation partners. Thus, it can be expected that the target numbers will be achieved, possibly even in a shorter recruitment time.

### 6.3.2 Sample Size Calculation for WIdO / DGU Trauma Register

In 2019, approximately 11,000 insured individuals aged 18-55 with a severe injury treated in an acute hospital were identified based on the existing identification criteria in the nationwide WIdO data. Over a three-year inclusion period, around 33,000 severely and critically injured insured individuals will be included in the study. With this target sample size, precise estimates of prevalences are also possible within small subgroups. For instance, in a subgroup of 5% of the total number of patients, a prevalence of 50% could be estimated with a precision of ±1.20% to 1.38% (two-sided exact binomial confidence interval 95% confidence interval). A prevalence of 50% was chosen as it shows the greatest variation. Higher or lower prevalences, as well as larger subgroups, would lead to a more precise estimate.

## 6.4 Recruitment Measures for the Prospective Cohort Study

The recruitment of severely and critically injured patients will take place through clinics in the DGU Trauma Network during their stay in the study clinic. The study clinics will receive comprehensive training on the study procedure and the follow-up surveys (processes, content). The study clinics are certified DGU trauma centers and have extensive experience in treating severely injured patients, treating 50-250 patients per year according to the study's inclusion criteria (see above).

## 7. Methodology and Implementation

To address the research questions, the project pursues a quantitative approach consisting of two cohorts of severely and critically injured patients, complemented by qualitative methods. Cohort 1 is a multicenter, non-interventional, prospective study focusing on clinical data, patient-reported experiences (PREs), and patient-reported outcomes (PROs). In Cohort 2, the focus is on retrospective analysis. For this, GKV routine data from the Scientific Institute of the AOK (WIdO) will be analyzed regarding patient pathways up to their reintegration into the workforce.

Qualitative methods are described in the ethical application 1 – qualitative study arm; these serve as the basis for parts of the questionnaire used in the multicenter, non-interventional, prospective cohort study. This questionnaire will be supplemented in the baseline survey by the DGU Trauma Register standard questionnaire (attached) and in the follow-up by existing instruments for surveying severely and critically injured patients identified through literature research.

### 7.1 Data Sources

Figure 1 below illustrates the data sources used in the LeAf Trauma study. The patient and expert interviews listed there are described in detail in Ethical Application 1 – Qualitative Study Arm. The importance of these interviews for the other study arms is evident from the figure: Qualitative data play a significant role, particularly for the prospective cohort study and also for the analysis of secondary data from WIdO. Below, the three other data sources, their methodologies, and their interrelations are described in detail.

Figure 1: Data Sources

### 7.1.1 Preliminary to the Prospective Cohort Study

The sections of the questionnaire that emerged from the qualitative group interviews (described in Ethical Application 1 – Qualitative Study Arm) will be evaluated in a pretest for clarity and usability and may be adjusted before being used in patient care (Version 1.0). The pretest will be conducted with patients who have completed their treatment and participated in the interviews. The follow-up assessment processes will be developed based on the results of patient interviews, literature research, and discussions with stakeholders from previously conducted follow-up studies on severely and critically injured patients, taking into account data protection and technical conditions (see Ethical Application 1 – Qualitative Study Arm).

### 7.1.2 Prospective Cohort Study

All participating study clinics will receive at least two training sessions explaining the study and follow-up procedures in detail. The recruitment, administration, tracking, and follow-up throughout the entire study will be conducted by the respective study clinic, which will

receive a tiered compensation of up to €660.00 per patient based on the number of successfully documented follow-ups in the web-based study database. Documentation of all assessments will be conducted exclusively in a pseudonymized manner in the web-based study database. The assignment list will be managed by the respective study clinic for the included patients.

After obtaining informed consent for study participation, baseline data will be collected before discharge (clinical data, profession, situation before the accident – exact contents are based on the results of patient and expert interviews as described in Ethical Application 1, Qualitative Study Arm; further information can be found in the section on Data Collection).

## Follow-Up Assessments

Follow-up assessments will be conducted 6, 12, and 18 months after the trauma, including the PREM and PROM developed in the project (see Figure 2). The optimal follow-up assessment processes will be developed based on the results of patient interviews, literature research, and discussions with stakeholders from previously conducted follow-up studies on severely and critically injured patients, while considering data protection and technical conditions. For the follow-up, patients will be surveyed either by mail or by phone from the respective study clinics. They will not be summoned by the study clinics. In an initial work package of the overall project, a patient group-specific assessment methodology for follow-up will be developed. If postal surveys prove to be the best method of inquiry for this patient group, responses will be returned to the treating study clinic. Other partners will not have knowledge of the patients' identities.

Figure 2: Flowchart of the Prospective Cohort Study LeAf Trauma

*FU = Follow Up, GCS = Glasgow Coma Scale, MAIS = Maximum Abbreviated Injury Scale

## Interim Evaluation

An interim evaluation of the study results will take place (after the 6-month and 12-month follow-ups), where the results (content-wise) from the follow-ups will be analyzed and compared with the findings from the patient and expert interviews (see Ethics Application 1 – Qualitative Study Component). These results will be supplemented with insights from the routine data analysis (October 2022 to June 2023) and interdisciplinary evaluations of the same, involving intersectoral experts. This may lead to an expansion of the PREMs and PROMs used in the questionnaire (Development of Questionnaire Version 1.1), which will then be employed in the 18-month follow-up. The goal of the interim evaluation is to determine whether further aspects relevant to the recovery process, health-related quality of life, and the regaining of work capacity have emerged in discussions with patients that were not included in the previous questionnaire and can be added as "free text." This may result in the need to adjust the PROMs and PREMs. A revised version of the PROMs and PREMs is expected to be used in the 18-month follow-up.

Figure 3: Flow of the Prospective Cohort Study

After the follow-up period, the contributors from the study clinics will share their experiences regarding the data collection, patient feedback, and their personal assessment of the data collection processes through an online survey.

The evaluation of the prospective study will commence with the preparation of the analysis algorithms in the 4th project quarter (2023). Interim results will contribute to the temporal and content characterization of patient pathways and the care reality of severely and critically injured GKV patients.

### 7.1.3 WIdO Data

The secondary data from WIdO will be utilized to characterize the temporal and content-related post-hospital treatment pathways of severely and critically injured patients until the regaining of work capacity over three years. To identify severely and critically injured patients, their subsequent diseases and treatments, as well as their predictors in routine data, definitions must first be developed based on medical knowledge that can be operationalized in GKV routine data. These are currently being developed and will then be submitted to WIdO. Furthermore, unfavorable disease courses for trauma-specific subgroups will be characterized. In medical expert groups, possible outcomes in defined post-hospital periods will be marked as unfavorable courses, e.g., readmissions, infections, non-unions, reoperations. The regaining of work capacity as a primary outcome, along with unfavorable courses, will be analyzed for previously identified relevant subgroups concerning possible predictors.

Only WIdO will conduct the analysis of this cohort, and the other consortium partners will receive only anonymized aggregated data of the defined cohort. These data will not be linked with other project data and cannot be traced back to a specific patient. WIdO will utilize nationwide AOK data from the years 2015 to 2020 for this purpose.

### 7.1.4 TraumaRegister DGU

The data from the TraumaRegister DGU from the years 2015 to 2017 will be used for a more in-depth representation of the cohort, for example regarding injuries and representativeness. Many clinical data of severely and critically injured patients are documented in the TraumaRegister DGU. The application includes the standard form for collecting patients in the TraumaRegister DGU, which provides an overview of the data to be collected in the TraumaRegister DGU. The data from the TraumaRegister DGU can only be analyzed through a formal request. This procedure is regulated by the application and review process according to publication guidelines (as described here: https://www.traumaregister-dgu.de/forschung). The data will then only be incorporated into the project in anonymized aggregated form.

### 7.1.5 Data Collection for the Prospective Cohort Study (Baseline and Follow-Up)

Patients will be surveyed a total of four times in the prospective arm of the study (baseline, 6, 12, and 18 months follow-up). At the time of discharge from the study clinic, a baseline survey will be conducted, followed by the follow-up collection of Patient-Reported Outcomes (PROs) regarding health-related quality of life (hrQoL) and work ability (AF), as well as an evaluation of the treatment pathway experienced by the patient (Patient-Reported Experiences (PREs)). The collection of patient data will be conducted via a questionnaire. The questionnaire will consist of several modules. Validated questionnaires will be used whenever possible. The measurement instruments listed under "Assessment of Influences on Work Ability after Trauma (PROM)" and "Patient-Reported Experience Measures (PREMs)" will be selected based on the patient and expert interviews and supplemented with independently constructed questions. The completion of the questionnaire should not take longer than 30

minutes. Before the start of patient recruitment, the questionnaire will undergo a pretest and will be revised as necessary.

- **TraumaRegister DGU Standard Form** (only during baseline survey, see attached documents)
- **Assessment of Quality of Life after Trauma (PROMs)**
  - POLO Chart
    Pirente, N., Bouillon, B., Schäfer, B. et al. Systematic Development of a Measurement Instrument for Assessing Health-Related Quality of Life in Polytraumatized Patients: The Polytrauma Outcome (POLO) Chart. Unfallchirurg 105, 413–422 (2002). https://doi.org/10.1007/s00113-001-0348-5
- **Assessment of Influences on Work Ability after Trauma**
  - Health-related factors (physical and psychological)
  - Sociodemographic factors
  - Individual factors and personality structure
  - Rehabilitation-related factors
  - Workplace-related factors
  - Injury characteristics and information regarding the hospital stay
- **Patient-Reported Experiences (PREMs)**
  - Treatment and care in the acute clinic
  - Assessment of medical interfaces in the patient pathway
  - Description of organizational hurdles with authorities and institutions

It is expected that additional characteristics of patient experience in the treatment pathway will be highlighted in the patient and expert interviews; these will be considered in the construction of the questionnaire.

## 7.2 Statistical Analysis

- **Description of Progress**: All patients; description of the regaining of work ability (AF) and health-related quality of life over time (3 follow-up points; descriptive statistics).
- **Predictors for AF**: AF up to 18 months after trauma as a yes/no criterion; univariate and multivariate analysis (logistic regression) of possible predictors; classification of predictors into the categories: patient, profession, pre-hospital, clinic, post-acute therapy, presence of psychological problems (from QoL assessment at follow-up time).
- **Predictors for Time until AF**: Only patients with regaining AF: Time until AF as a dependent variable, Kaplan-Meier curves for description, Cox regression for multivariate analysis, predictors as above.
- **Health-related Quality of Life and PREMs**: Description at the 3 follow-up points, correlation with AF as subgroup comparisons at each measurement time.

## 8. Voluntariness and Withdrawal

Participation in the study is voluntary for patients. Consent can be withdrawn by participants at any time, without providing reasons and without personal disadvantages, either in whole or in part. Before the study begins, participants will be informed both in writing and orally about the study procedure and data protection. Their consent will be documented by signature on the

consent form. In the event of withdrawal from the study, any data already collected will be destroyed unless the participant agrees to the evaluation of that data.

When informing patients and obtaining consent, they will also be asked to agree to the scientific secondary use of the study data collected in the context of the LeAf Trauma project. This consent is optional and has no impact on participation in the prospective multicentric cohort study.

Since all participating study clinics are certified Trauma Centers DGU, they are contractually obligated to include patients who meet the inclusion criteria of the TraumaRegister DGU and have given their consent in the TraumaRegister DGU. Therefore, patients who meet the inclusion criteria for both the prospective LeAf Trauma cohort study and the TraumaRegister DGU will also be asked for their optional consent to participate in the TraumaRegister DGU. Only routine documentation data from the clinic will be recorded in the TraumaRegister. No additional data will be collected. Participation in the TraumaRegister is voluntary and does not affect the possibility of participating in the LeAf Trauma study.

## 9. Adverse Effects and Burdens for Participants

The processing and use of primary data from the prospective cohort study will be conducted with consideration of integrity (e.g., protection against intentional or negligent falsification of programs or data manipulation), confidentiality (e.g., protection against unauthorized access to data), and availability (e.g., protection against theft or destruction). Another central point is the necessity of pseudonymizing data. The protection of patient data will be ensured through current technical and organizational protective measures, as outlined in the data protection concept for the prospective cohort study. Thus, the data protection risks are considered low.

There is a possible psychological burden from actively recalling the trauma/recovery process; however, the participating study clinics have extensive experience in treating severely and critically injured patients (between 40 and 250 patients annually). Furthermore, the project's scientific staff are aware of the sensitivity of the discussion topic and will inform and train the participating study clinics on this issue during the planned training sessions.

## 10. Benefit-Risk Analysis

The personal benefit from participation is considered very low; however, the time commitment for patients is also minimal. There is a potential risk for psychological strain on the patients, as explained under "Complications." On the other hand, there is significant potential for the utilization of the insights gained in various respects, such as:

- The indispensability of the results in developing recommendations for improving the care of severely and critically injured patients – this directly addresses the reality of care and aims to identify and possibly eliminate barriers to re-entering working life, thereby improving care.
- The significance of the results for all involved in the care of severely and critically injured patients, as the findings will raise awareness of quality issues in care, optimize care pathways, and pave the way for new collaborations – both interprofessional and intersectoral.
- Improvement measures in the acute phase of treatment for severely and critically injured patients should be directly incorporated into accompanying projects and initiatives of the DGU – for example, the certification of Trauma Centers and Trauma

Networks DGU, as well as cooperation between Trauma Networks and rehabilitation clinics.

- The general relevance of the results for training, further education, and continued education of professionals involved in the care of severely and critically injured patients.
- Improvement of care for future severely and critically injured patients.

## 11. Data Management and Data Protection

Patient data will be stored in a central study register. Data transmission to the study register occurs via a web-based secured connection (https) after login with a username and password by the involved medical personnel. The operator of the study register is the project management AUC. Data processing follows a detailed rights and role concept. Details can be found in the data protection concept, which is located in the attachments. A differentiated consent management system with established documentation concepts ensures transparency and information about the ability to intervene. Patients can express their rights as affected individuals at any time to the study management or to any other consortium partner as the responsible party or to the treating study clinic. The data collection and processing includes a tiered role and access rights concept based on the principle of necessity, following an identity management application. The authorization of initial users of the LeAf Trauma study register from the study clinics occurs after contract signing via the AUC. User authentication for the study register is performed using a username and password. The personnel involved are limited to verifiably responsible, qualified, and formally authorized individuals. They are contractually or professionally obligated to maintain data confidentiality. A secure authentication procedure using a username and password is implemented.

Fig. 4: Data Flows and Processing Steps in the Prospective Cohort Study within the LeAf Trauma Project
