# Supplementary material for: LeAf Trauma- an intersectoral prospective multicenter study assessing quality of life and return to work after majortrauma–study protocol
Source: PLoS One. 2024 Nov 13;19(11):e0312320. doi: 10.1371/journal.pone.0312320 (PMC11560036; doi:10.1371/journal.pone.0312320)
Supplement: S6 File — (PDF) [file pone.0312320.s006.pdf]

S6 -WHO Trial Registration Data Set

(Version 1.3.1)

1. **Primary Registry and Trial Identifying Number**: German Clinical Trials Register (DRKS): DRKS00028841

2. **Date of Registration in Primary Registry**: 10/25/2022

3. **Secondary Identifying Numbers**
   Study No.: 2022-2029 (Ethics Committee at the Faculty of Medicine, Department of Orthopedics and Trauma Surgery, UKD)

   Funding Code: 01VSF21033 (Innovation Fund of the Federal Joint Committee (G-BA))

4. **Source(s) of Monetary or Material Support**: Innovation Fund of the Federal Joint Committee (G-BA)

5. **Primary Sponsor:** n.a.
   AUC-Akademie der Unfallchirurgie

6. **Secondary Sponsor(s):** n.a.
   German Trauma Society e.V., Section NIS

7. **Contact for Public Queries**
   Christine Hoefer
   Akademie der Unfallchirurgie, Emil-Riedel-Str. 5, 80538 Munich, Germany
   Phone: +4989 5404810
   Email: christine.hoefer@auc-online.de

8. **Contact for Scientific Queries**
   Katharina Fetz and Rolf Lefering
   Institute for Research in Operative Medicine,
   Witten/Herdecke University,
   Ostmerheimer Straße 200, Cologne, Germany

9. **Public Title**: Quality of life and return to work after major trauma.

10. **Scientific Title**: LeAf Trauma - an intersectoral prospective multicenter study assessing quality of life and return to work after major trauma - Study protocol

11. **Countries of Recruitment**: Germany, Austria

12. **Health Condition(s) or Problem(s) Studied**: n.a.

13. **Intervention(s)**: no interventions

14. **Key Inclusion and Exclusion Criteria**: See Inclusion and Exclusion Criteria in manuscript**.**

15. **Study Type**: This paper describes the study protocol of an intersectoral prospective multicenter cohort study.

16. **Date of First Enrollment**: Inclusion of patients started in 12/2022.

17. **Sample Size:** Sample Size consists of 1500 patients; inclusion is still ongoing.

18. **Recruitment Status**: Recruiting - participants are currently being recruited and enrolled.

19. **Primary Outcome(s)**: Primary outcomes are “Return to work” and “Health related quality of life” Please see figure 1.

20. **Key Secondary Outcomes**: Secondary outcomes are PROMs and PREMS, please see figure 1 and table 1.

21. **Ethics Review**
    Ethics Committee at the Faculty of Medicine
    Department of Orthopedics and Trauma Surgery, UKD
    Prof. Dr. med. Joachim Windolf
    Moorenstr 5
    40225 Düsseldorf

    Study No.: 2022-2029-other research first voting
    LeAf Trauma - Quality of Life and Work Ability After Severe Trauma - Quantitative Study Arm
    Date: 10/17/2022

22. **Completion date**: Date of study completion is 12/31/2025

23. **Summary Results**: no results yet.

24. **IPD sharing statement**: There is no plan to share IPD. Research by third parties with disclosure of raw data is not planned and not the subject of the consent obtained. Scientific reuse however is possible in cooperation and with sole access to the raw data by the AUC.
