# Supplementary material for: LeAf Trauma- an intersectoral prospective multicenter study assessing quality of life and return to work after majortrauma–study protocol
Source: PLoS One. 2024 Nov 13;19(11):e0312320. doi: 10.1371/journal.pone.0312320 (PMC11560036; doi:10.1371/journal.pone.0312320)
Supplement: S7 File — (PDF) [file pone.0312320.s007.pdf]

S7: Itemset list- translated in English

**subject**	**instrument**		
medical details	ICD-10-Code (1)		-
	self-constructed questions		**Please describe the pre-existing conditions of the patient and check the applicable options. (Multiple answers possible)**<br>**Common secondary diagnoses**<br>• Nicotine abuse<br>• Therapeutic anticoagulation<br>• Obesity<br>• Chronic alcohol abuse (not every alcohol consumption after a fall)<br>• Medication/drug abuse<br>• Chronic wounds<br>**Heart/Circulation**<br>• Heart failure<br>• Coronary heart disease<br>• Cardiovascular diseases<br>• Absolute arrhythmia<br>• Respiratory diseases (e.g., COPD, asthma)<br>**Metabolic diseases/Gastroenterology**<br>• Thyroid diseases<br>• Diabetes mellitus<br>• Kidney diseases<br>• Stomach/intestinal/liver/gallbladder diseases<br>**Neurology/Psychiatry**<br>• Neurological diseases<br>• Psychiatric/psychosomatic diseases<br>**Other chronic diseases**<br>• Chronic infectious diseases (e.g., HIV, tuberculosis)<br>• Malignant diseases<br>• Chronic rheumatic diseases<br>• Chronic pain disorders
			**Please indicate the payer for the patient's treatment.**<br>• Privately insured<br>• Privately additionally insured (for the clinic)<br>• Statutory health insurance<br>• Occupational accident insurance<br>• Other (self-payer)
			**Did the patient already have pain therapy as home medication upon admission?"**<br>• Yes<br>• No

			**What additional pain therapy did the patient receive (if any) in addition to their home medication? (Multiple answers possible)"**<br>**Peripheral (WHO step 1) (yes/no)**<br>• NSAIDs<br>• Metamizole<br>• Paracetamol<br>**Opioids (yes/no)**<br>• Tramadol<br>• Tilidine (with or without naloxone)<br>• Hydromorphone<br>• Oxycodone<br>• Morphine (MST/Sevredol)<br>• Transdermal/patch<br>**Co-analgesics (yes/no)**<br>• Carbamazepine<br>• Gabapentin<br>• Pregabalin<br>• Amitriptyline<br>• Clomipramine<br>• Duloxetine<br>• Venlafaxine
			**The patient's discharge will take place...**<br>Timely = within the first 2 weeks after discharge<br>• To home (with or without physical therapy)<br>• To home, with the timely start of outpatient rehabilitation<br>• To home, with the timely start of inpatient rehabilitation<br>• Transfer to an inpatient rehabilitation facility<br>• Transfer to an occupational accident insurance inpatient follow-up treatment (BGSW) / complex inpatient rehabilitation (KSR)<br>• Transfer to a care facility for short-term care<br>• Transfer to a permanent care facility<br>• Transfer to another hospital<br>• Other
	Barthel-Index, Assessment of basic activities of daily living (2)		-
	Trauma-Reha-Score Screening (3)9/20/2024 8:33:00 AM		-
	Trauma-Register data (4)		-
person	body mass index		**Your height: _______ cm**<br>**Your weight before the accident: _______ kg**
	self-constructed questions		**How old are you?**
			**Your gender:** ☐ male ☐ female ☐ diverse

			**Please indicate your marital status:**<br>☐ single<br>☐ in a partnership<br>☐ married / registered civil partnership<br>☐ divorced / registered civil partnership dissolved<br>☐ widowed / registered partner deceased
			**Have you or your parents immigrated to Germany from abroad?**<br>☐ Yes, I myself<br>☐ Yes, my parents<br>☐ No
			**Were you unable to work due to illness in the last calendar year before the accident?** If yes, how many weeks: _________ weeks.
social support	Oslo Social Support Scale (5)		-
	self-constructed question		**With the following statements, we want to assess the impact of the accident on your current well-being. Please consider only the past 4 weeks when answering. Check what applies.**<br>• My friendship/partnership/marriage has suffered because of the accident.<br>• I do not feel well supported by my close environment (friends/relatives).<br>• The contact with my close environment (friends/relatives) has worsened.<br>**Response options:**<br>• Applies<br>• Largely applies<br>• Partially applies<br>• Barely applies<br>• Does not apply
			**Have you experienced any separations or losses of close individuals in the last 18 months after the accident?**<br>• Separation from partner<br>• Loss of close friends<br>• No
social responsibility	*self-constructed questions*		**How many people are in your household? _________ people**<br>This question refers to all adults and children in your household, including yourself, who live off the shared household income.
			**Are you the main earner of the household?**<br>• Yes<br>• No<br>• Two roughly equal incomes in the household
			**With the following statements, we want to assess the impact of the accident on your current well-being. Please consider only the past 4 weeks when answering. Check what applies**<br>• I have professional/financial problems.<br>**Response options:**<br>• Applies<br>• Largely applies<br>• Partially applies<br>• Barely applies<br>• Does not apply

social status	Winkler-Index<br>(6);(7)		**What is your current monthly net household income? This refers to the total income of all persons in the shared household.**<br>• less than 1250 €<br>• 1250 € to 2500 €<br>• more than 2500 €
			**Please try to classify your highest educational qualification:**<br>☐ No school leaving certificate<br>☐ Secondary school certificate (qualifying degree)<br>☐ Realschulabschluss (intermediate school leaving certificate)<br>☐ Technical college entrance qualification<br>☐ Gymnasium (Abitur)<br>☐ Completed vocational training<br>☐ University degree (Bachelor, Master craftsman)<br>☐ University degree (Master, Diploma, State examination)
motivation of rehabilitation	PARMEO, Patient questionnaire for assessing rehabilitation motivation (8)	level of information	-
		scepticism	-
profession	ISCO, international scale of occupation (9)		**Please assign your current occupation/training to one of the listed job categories:**<br>☐ Executive, office work (e.g., management, project leader)<br>☐ Executive, craft sector (e.g., head of a craft business, project leader)<br>☐ Police, armed forces<br>☐ Professional sports<br>☐ Office work (e.g., financial planning, insurance, construction planning)<br>☐ Non-craft service profession (e.g., train attendant, salesperson, mail carrier)<br>☐ Craft or industrial work with heavy physical demands (e.g., tiler, scaffolder, carpenter)<br>☐ Craft or industrial work with physical activity (e.g., technician, hairdresser)<br>☐ Fine craft (e.g., goldsmith, musician)<br>☐ Social profession (e.g., kindergarten or teaching staff)<br>☐ Medical personnel (e.g., nursing, physiotherapy, doctors, MTA)<br>☐ Scientist<br>☐ Artist, journalist (e.g., author, actor)<br>☐ Agriculture and fishing<br>☐ Other
	REFA (German Committee for Determining Working Hours), Classification of heavy workload (10)		**Which statement applies to the physical strain at your workplace?**<br>• Work without any particular (physical) strain<br>• Handling light workpieces and tools, carrying less than 10 kg<br>• Climbing stairs and ladders, lifting and carrying medium loads on flat ground (about 10-15 kg), walking long distances on foot<br>• Tasks such as carrying loads of about 20-40 kg on flat ground, medium-heavy work in tense postures, e.g., kneeling<br>• Tasks such as lifting and carrying loads over 50 kg, heavy work in tense postures, e.g., kneeling
	self-constructed questions		**Which of the following categories best describes your employment status before the accident? I am...**<br>• Worker

			• Employed<br>• Self-employed, freelance<br>• Civil servant<br>• Seeking employment<br>• In training/education<br>• Other
			**Were you employed before the accident?**<br>• Yes, full-time (35 or more hours per week)<br>• Yes, part-time, between 15 and 34 hours per week<br>• Yes, part-time, less than 15 hours per week<br>• No, homemaker<br>• No, in education/training<br>• No, unemployed/seeking work<br>• No, early retired<br>• No, currently on leave
general work motivation	DIAMO, Diagnostic instrument for work motivation (11)	attitude regarding work	-
		goal-inhibition	-
		goal-activity	-
sellf-appraisal of injury severity	*self-constructed questions*		**How severe was the accident for you, on a scale from 0 to 10?**<br>0 = not severe at all, 10 = very severe
			**Do you believe you will be able to work in your profession and perform your duties as before the accident?**<br>• Yes, I expect to be able to fully resume my profession and duties as before the accident.<br>• Yes, I expect to be able to resume my profession and duties as before the accident, but with some limitations.<br>• I cannot assess this at the moment.<br>• No, I do not expect to be able to perform my previous duties, but I can likely take on an alternative role within my profession.<br>• No, I do not expect to be able to work in my profession or in a similar field.
			**Do you believe that you could have avoided the accident yourself in that situation?"**<br>• Yes<br>• Probably<br>• Probably not<br>• No<br>• I don't know
self-efficacy and resilience	ASKU, German General Self-Efficacy Short Scale (12)		-
	Re-Re Scale Resistance-Regeneration Orientation Scale (13)		-
health related quality of life	European Quality of Life 5 Dimensions 3 Level Version (14)		-

	TOP, Trauma Outcome Profile (15)	anxiousness	-
		depression	-
		posttraumatic stress disorder	-
		pain	-
		body functions	-
		social interaction/ financial problems	-
		activity of daily living (ADL)	-
		mental function	-
		body image	-
		overall satisfaction	-
	Short-Form-Health-Survey-12 (16)		-
	self-constructed questions		**How are you?**<br>Please mark a value on the scale from 0 to 10.<br>0 means "very poor" and 10 means "very good."
			**How would you describe your current sleep quality?**<br>• Very good<br>• Good<br>• Neither good nor bad<br>• Bad<br>• Very bad
			**How much does your religion/faith/spirituality help you in coping with the consequences of your accident?**<br>0 = "not at all"<br>10 = "very much"
			**How has the quality of your sex life changed at present compared to before the accident?"**<br>-5 = very negatively<br>0 = neutral<br>5 = very positively
circumstances of accident	*self-constructed questions*		**In what context did your accident occur?**<br>• Sports<br>• Other leisure activity (not sports)<br>• Work / Work commute / Training<br>• Household
			**Do you remember the accident?**<br>• Yes<br>• No
			**What applies to your accident?**<br>• Single-vehicle accident (no other persons involved)<br>• There were other participants (uninjured)<br>• There were other injured persons
			**How severe was the accident for you, on a scale from 0 to 10?**<br>0 = not severe at all, 10 = very severe

			**Do you believe that you could have avoided the accident yourself in that situation?**<br>• Yes<br>• Probably<br>• Probably not<br>• No<br>• I don't know
			**Are you currently involved in a legal procedure related to the accident? (e.g., with the other party involved in the accident)**<br>• Yes<br>• No
			**Regardless of who caused the accident, do you feel guilty about the accident? This is about your personal experience, not legal responsibility.**<br>• Yes<br>• Probably<br>• Probably not<br>• No<br>• I don't know
perception of acute care treatment	*self-constructed questions*		**How well did you feel you were treated in the emergency situation? (This refers to the situation before reaching the hospital.)**<br>• Very well<br>• Well<br>• Average<br>• Poor<br>• Very poor<br>• I do not remember the emergency situation
			**How well did you feel you were treated in the hospital's emergency room?**<br>• Very well<br>• Well<br>• Average<br>• Poor<br>• Very poor<br>• I do not remember the emergency room or shock room
			**How well informed did you feel about your treatment at the current hospital? (excluding the emergency room)**<br>• Very well<br>• Well<br>• Average<br>• Poor<br>• Very poor
			**How effective was the pain management at the current hospital for you? Please mark the appropriate value.**<br>My pain was treated 0 = very poorly 10 = very well.
return to work	*self-constructed questions*		**Do you know the current payer of your treatment?**<br>• Private health insurance<br>• Statutory health insurance/pension insurance

			• Occupational accident insurance<br>• Liability insurance of the other party involved in the accident<br>• Other payer<br>• I do not know the payer.
			**Are you currently unable to work?**<br>• Yes<br>• No<br>• I am currently in a reintegration program<br>• I am currently in retraining<br><br>**When did your inability to work end? Please provide the date.**
			**Have you had a gradual reintegration into work?**<br>• Yes<br>• No
			**Has there been a change in your income compared to the time before the accident?**<br>• Yes, I have a higher income<br>• Yes, I have a lower income<br>• No<br><br>**Are you working in the same position with reduced hours?**<br>• Yes, part-time, between 15 and 34 hours per week<br>• Yes, part-time, less than 15 hours per week<br>• No
			**Have you undergone retraining?**<br>• Yes<br>• No
			**Are you currently working to the same extent, in the same position, and with the same salary as before the accident?**<br>• Yes<br>• No
			**Please describe your current situation.**<br>• Homemaker<br>• Unemployed / seeking work<br>• Early retirement<br>• Currently on leave
			**How do you commute to work?**<br>**Or, how did you commute before the accident, if you are currently unable to work?**<br>• **I rely / relied on a car.**<br>• **I rely / relied on public transportation.**<br>• **I can / could (partially) work from home.**<br>• **Other (e.g., carpool, walking)**
			**Do you agree with the statement: The support from the employer means a lot to me?**<br>• Applies

			• Largely applies<br>• Partially applies<br>• Barely applies<br>• Does not apply
rehabilitation	*self-constructed questions*		**Where are you currently located?**<br>• Clinic / Hospital<br>• Rehabilitation facility (outpatient/inpatient)<br>• Care facility<br>• At home, working<br>• At home, in reintegration/retraining<br>• At home, on sick leave
			**Did you feel well informed about the further treatment upon discharge from the hospital?**<br>• Yes<br>• Partially<br>• No
			**Have you undergone rehabilitation (rehab)?**<br>• Yes<br>• No<br>**Was the rehabilitation conducted inpatient or outpatient?**<br>• Inpatient<br>• Outpatient
			**Were you transferred from the hospital directly or within two weeks to the inpatient rehabilitation?**<br>• Yes<br>• No
			**Please provide the date when the rehabilitation began.**<br>**Please provide the date when the rehabilitation ended.**<br>• Date:<br>• The rehabilitation is still ongoing.
			**Did you have a rehabilitation program specifically tailored to your accident-related issues?**<br>• Yes, the program was tailored to me<br>• Partially, the program was mostly general and not specifically for my injury consequences<br>• No, the program was almost exclusively for groups and not tailored to my specific accident consequences
			**Were you able to participate in the rehabilitation offerings?"**<br>• Yes<br>• Partially<br>• No
			**Did you experience improvement after the rehabilitation?**<br>• Yes<br>• Partially<br>• No

			**Did you receive the necessary support, including all aids and care assistance, for discharge from rehabilitation to home?**<br>• Yes<br>• Partially<br>• No<br>• I was transferred to a care facility.<br>• I am still undergoing rehabilitation.
			**Were you supported throughout the entire healing process by a central contact person (non-medical staff)? (Multiple answers possible)**<br>• Yes, by a rehabilitation manager<br>• Yes, by an occupational therapist<br>• Yes, by a dedicated case manager from my health insurance/occupational accident insurance<br>• No<br><br>**"Did you find this support helpful?"**<br>• Yes<br>• Partially<br>• No
secondary gain of trauma	FPTM - Questionnaire for assessing psychotherapy motivation<br>(17)	symptom-focused attention from others	-
body shame	*self-constructed questions*		**To what extent do you agree with the following statements? (0 = not at all, 5 = very much)**<br>1. Since the accident, I am less comfortable showing my body.<br>2. Since the accident, I have physical limitations that make me uncomfortable.<br>3. Since the accident, I consciously hide parts/areas of my body.<br>4. Since the accident, I feel less comfortable in my body.<br>5. Since the accident, I feel less attractive.<br>6. Since the accident, I dress differently.<br>7. Since the accident, I cover skin areas/scars with makeup/cloths
post hospital treatment	*self-constructed questions*		**Did you receive the necessary support, including all aids and care assistance, for discharge from the hospital to home on the day of discharge?"**<br>• Yes<br>• Partially<br>• No<br>• I was transferred to a care facility.
			**Did you ever feel helpless during the time between discharge and rehabilitation?"**<br>Please rate from 0 to 10, where 0 means "not at all" and 10 means "extremely."
			**Did you always feel well informed about the further treatment?**<br>0 = very poorly<br>10 = very well
			**Did you receive physiotherapy/occupational therapy after discharge from the hospital and before starting rehabilitation?"**

			• Yes<br>• No
			**Which doctor has treated the consequences of your accident and organized/prescribed further therapy after discharge from the hospital and the rehabilitation clinic?**<br>• General practitioner<br>• Orthopedist<br>• Trauma surgeon<br>• General surgeon<br>• Outpatient rehabilitation doctor<br>• Not applicable
			**Please rate the coordination of the treatment process by the attending doctor.**<br>0 = very poor, 10 = very good
			**Please describe the pain management after discharge from the hospital. How well were your pains treated? This applies to all patients, with or without rehabilitation, who are currently not in the hospital."**<br>0 = very poorly, 10 = very well<br>I did not require pain management
			**Since your discharge from the hospital, have you been in psychological/psychotherapeutic/psychiatric treatment due to the consequences of the accident?**<br>• Yes<br>• No
			**In your opinion, is the support provided by health services such as home care, physiotherapy, occupational therapy, or aids sufficient?**<br>• Yes<br>• No
			**Have you ever had problems dealing with authorities, such as issues related to retraining or the payment of sickness benefits, unemployment benefits, etc.?**<br>• Yes<br>• No
			**How well did you feel treated in the acute hospital?**<br>0 = very poorly, 10 = very well
			**How well did you feel treated in the outpatient setting?**<br>0 = very poorly, 10 = very well
medical courses of trauma	*self-constructed questions*		**Did you experience any complications during the healing process? (e.g., infection, delayed fracture healing, fall)**<br>• Yes<br>• No
			**Were any additional surgical procedures performed after discharge from the hospital?**<br>• Yes<br>• No
			**Has a new illness been diagnosed since the accident?**<br>• Yes

			• No
personality	Big Five Factor Model (18)		-
PTSD - posttraumatic stress dissorder	International trauma questionnaire (19)		-
treatment evaluation	*self-constructed questions*		**Are you overall satisfied with the treatment outcome after the accident?**<br>• Better than expected<br>• As expected<br>• Worse than expected
			**How do you perceive your overall life situation after the accident?**<br>• Better than expected<br>• As expected<br>• Worse than expected
			**To what extent has the accident event positively or negatively changed your view on life?**<br>-5 = very negative<br>0 = neutral<br>5 = very positive
			**Have you noticed any problems with the care provided? (Multiple answers possible)**<br>• Waiting time for physiotherapy/manual lymphatic drainage<br>• Number of physiotherapy/manual lymphatic drainage appointments<br>• Psychotherapy<br>• Waiting time for inpatient rehabilitation measures<br>• Provision of aids<br>• Duration until aids were available<br>• No, I have not noticed any problems<br>• Not applicable to me

1. The Lancet null. ICD-11. Lancet Lond Engl. 8. Juni 2019;393(10188):2275.
2. Collin C, Wade DT, Davies S, Horne V. The Barthel ADL Index: a reliability study. Int Disabil Stud. 1988;10(2):61–3.
3. Rosenhauer V, Simmel S. [Trauma reha score : How can we define the rehabilitation needs of the severely injured?]. Unfallchirurg. Mai 2021;124(5):419–26.
4. Trentzsch H, Maegele M, Nienaber U, Paffrath T, Lefering R. Der Datensatz des TraumaRegister DGU®, seine Entwicklung über 25 Jahre und Fortschritte in der Schwerverletzenversorgung. Unfallchirurg. Oktober 2018;121(10):794–801.
5. Kocalevent RD, Berg L, Beutel ME, Hinz A, Zenger M, Härter M, u. a. Social support in the general population: standardization of the Oslo social support scale (OSSS-3). BMC Psychol. 17. Juli 2018;6(1):31.

6. Winkler J, Stolzenberg H. [Social class index in the Federal Health Survey]. Gesundheitswesen Bundesverb Arzte Offentlichen Gesundheitsdienstes Ger. Dezember 1999;61 Spec No:S178-183.

7. Lampert T, Kroll LE, Müters S, Stolzenberg H. Messung des sozioökonomischen Status in der Studie „Gesundheit in Deutschland aktuell" (GEDA). Bundesgesundheitsblatt - Gesundheitsforschung - Gesundheitsschutz. Januar 2013;56(1):131–43.

8. Hafen K, Jastrebow J, Nübling R, Bengel J. [Development of a patient questionnaire for assessment of motivation for rehabilitation(PAREMO)]. Rehabil. Februar 2001;40(1):3–11.

9. Hoffmeyer-Zlotnik J, Hess D, Geis A. Computerunterstützte Vercodung der International Standard Classification of Occupations (ISCO-88). ZUMA Nachrichten. 1. Januar 2004;

10. Abele CR. Arbeitsunfähigkeit und Minderung der Erwerbsfähigkeit in Abhängigkeit der Arbeitsschwere nach der REFA-Klassifikation und dem Typ der Tibiakopffraktur [Internet] [Dissertation]. Universität Tübingen; 2017 [zitiert 22. Mai 2022]. Verfügbar unter: https://publikationen.uni-tuebingen.de/xmlui/handle/10900/78033

11. Ranft A, Fiedler R, Greitemann B, Heuft G. [Diagnostics of work motivation (DIAMO): optimization and construct validity]. Psychother Psychosom Med Psychol. Januar 2009;59(1):21–30.

12. Beierlein C, Kovaleva A, Kemper CJ, Rammstedt B. ASKU - Allgemeine Selbstwirksamkeit Kurzskala. 2012 [zitiert 15. Mai 2024]; Verfügbar unter: https://www.psycharchives.org/jspui/handle/20.500.12034/431.2

13. Otto J, Linden M. ReRe-Skala - Resistenzorientierung-Regenerationsorientierungs-Skala. 2018 [zitiert 15. Mai 2024]; Verfügbar unter: https://www.psycharchives.org/handle/20.500.12034/623.2

14. Balestroni G, Bertolotti G. EuroQol-5D (EQ-5D): an instrument for measuring quality of life. Monaldi Arch Chest Dis [Internet]. 2012 [zitiert 5. Dezember 2023];78(3). Verfügbar unter: https://www.monaldi-archives.org/index.php/macd/article/view/121

15. Gross T, Amsler F, Attenberger C. Evaluation des Trauma Outcome Profile (TOP) im Langzeitverlauf nach Polytrauma. In German Medical Science GMS Publishing House; 2011. S. DocWI60-1015.

16. K D, M M, H G, E B, Ma W. [Validation of the Short-Form-Health-Survey-12 (SF-12 Version 2.0) assessing health-related quality of life in a normative German sample]. Z Psychosom Med Psychother [Internet]. September 2020 [zitiert 18. Mai 2022];66(3). Verfügbar unter: https://pubmed-1ncbi-1nlm-1nih-1gov-100a4a9v50105.han.med.uni-magdeburg.de/32876561/

17. Schulz H, Lang K, Nübling R, Koch U. Psychometrische Überprüfung einer Kurzformdes Fragebogens zur Psychotherapiemotivation - FPTM-23. Diagnostica. 1. April 2003;49:83–93.

18. Caprara GV, Barbaranelli C, Borgogni L, Perugini M. The "big five questionnaire": A new questionnaire to assess the five factor model. Personal Individ Differ. 1. September 1993;15(3):281–8.

19. Cloitre M, Shevlin M, Brewin CR, Bisson JI, Roberts NP, Maercker A, u. a. The International Trauma Questionnaire: development of a self-report measure of ICD-11 PTSD and complex PTSD. Acta Psychiatr Scand. Dezember 2018;138(6):536–46.
